# A Quantitative Review of Urban Ecosystem Service Assessments: Concepts, Models, and Implementation

**DOI:** 10.1007/s13280-014-0504-0

**Published:** 2014-04-17

**Authors:** Dagmar Haase, Neele Larondelle, Erik Andersson, Martina Artmann, Sara Borgström, Jürgen Breuste, Erik Gomez-Baggethun, Åsa Gren, Zoé Hamstead, Rieke Hansen, Nadja Kabisch, Peleg Kremer, Johannes Langemeyer, Emily Lorance Rall, Timon McPhearson, Stephan Pauleit, Salman Qureshi, Nina Schwarz, Annette Voigt, Daniel Wurster, Thomas Elmqvist

**Affiliations:** 1 Humboldt Universität zu Berlin, Berlin, Germany; 2 Helmholtz Centre for Environmental Research – UFZ, Leipzig, Germany; 3 Stockholm Resilience Centre, Stockholm, Sweden; 4 Paris Lodron University of Salzburg, Salzburg, Austria; 5 Universitá Autonomá de Barcelona, Barcelona, Spain; 6 Beijer Institute of Ecological Economics in Stockholm, Stockholm, Sweden; 7 Milano School of International Affairs, Management and Urban Policy, The New School, New York, NY USA; 8 Technical University of Munich, Munich, Germany; 9 Tishman Environment and Design Center, The New School, New York, NY USA

**Keywords:** Review, Urban ecosystem services, Models, Demand-provisioning, Policy implementation

## Abstract

Although a number of comprehensive reviews have examined global ecosystem services (ES), few have focused on studies that assess urban ecosystem services (UES). Given that more than half of the world’s population lives in cities, understanding the dualism of the provision of and need for UES is of critical importance. Which UES are the focus of research, and what types of urban land use are examined? Are models or decision support systems used to assess the provision of UES? Are trade-offs considered? Do studies of UES engage stakeholders? To address these questions, we analyzed 217 papers derived from an ISI Web of Knowledge search using a set of standardized criteria. The results indicate that most UES studies have been undertaken in Europe, North America, and China, at city scale. Assessment methods involve bio-physical models, Geographical Information Systems, and valuation, but few study findings have been implemented as land use policy.

**Electronic supplementary material:**

The online version of this article (doi:10.1007/s13280-014-0504-0) contains supplementary material, which is available to authorized users.

## Introduction

### The Global Urban Dimension

Cities are complex adaptive systems embedded within even more complex adaptive ecosystems (Burkhard et al. 2010). Cites and their regions are hubs for people, infrastructure and commerce, requiring extensive resources and putting intense pressure on the environment (Grimm et al. 2008). Urban landscapes are the everyday environment of the majority of the global population (>51 %), including nearly 80 % of European and US citizens, almost 50 % of Asians and >90 % of Latin Americans (UN 2012; Haase 2014). The continuous increase in the number and size of cities and the ensuing transformation of virgin landscapes on different scales pose significant challenges for reducing the rate of biodiversity loss and related ecosystem functionality and ensuring human welfare. Plants, animals, and microorganisms, that is, biodiversity, is the basis of all ecosystems and the services they provide. Because urbanisation and soil sealing provoke changes, predominantly a decline, in species diversity and human well-being in cities both “…are inextricably linked” (Millennium Ecosystem Assessment 2005b).

However, urban areas also provide a range of benefits to sustain and improve human livelihood and the quality of life through urban ecosystem services, UES (TEEB 2011). UES have been classified in a variety of ways; most commonly, they are divided into four categories: provisioning services, regulating services, habitat or supporting services, and cultural services (Millennium Ecosystem Assessment 2005a; Cowling et al. 2008; TEEB 2011). Provisioning services include material outputs from ecosystems, including food, water, medicinal plants, and other resources. Regulating services maintain functions, such as air and soil quality and flood, storm water and disease control. Habitat and supporting services underpin almost all other services by providing living spaces for organisms. Supporting services also maintain plant and animal diversity. Finally, cultural services include the non-material, socio-ecological benefits (including psychological and cognitive benefits) people obtain from contact with environs, such as recreation, esthetic, spiritual, and psychological benefits and tourism (TEEB 2011). In general, locally generated ES have substantial impacts on the quality of life in urban areas and should, therefore, be more explicitly addressed in conceiving strategies aimed at sustainable development, liveability, and resilience in urban milieu (Gomez-Baggethun et al. 2013).

The Millennium Ecosystem Assessment concluded that 60 % of ES are degraded or used unsustainably, having adverse effects on human well-being (Millennium Ecosystem Assessment 2005a). Because almost no ecosystems remain un-impacted by humans and humans cannot exist without ecosystems, protection and sustainable use of ecosystems are no longer an isolated interest but a key component of global sustainable development. The observed rapid degradation of the ability of ecosystems to generate services not only necessitates a better understanding of how to maintain important ecosystem functions but also requires that this knowledge is put into a broad institutional and governance context (TEEB 2011). To address the challenges of ecosystem degradation, an interdisciplinary social–ecological system approach is critically important and needed at this time (Folke et al. 2004).

Today, cities are facing enormous challenges, such as climate change, demographic aging, and natural resource depletion. Ecosystems play an important role in facilitating transformations needed to address these challenges. Understanding how urban ecosystems work, how they change, and what limits their performance can add to the general understanding of ecosystem change and governance in an ever more human-dominated world (Elmqvist et al. 2008). In general, functioning ecosystems provide the flexibility in urban landscapes to build adaptive capacity and cope with problems such as increased risks of heat waves and flooding. Although urban social–ecological system analyses have been found to be promising for enhancing our understanding of how exactly ecosystems can help address the moderation of climate change effects, large knowledge gaps, particularly for cities, are still present. For example, urban ecosystems were vastly under-represented in the world’s largest assessment of ecosystems. The TEEB study (2011) made one of the first successful attempts to explicitly represent urban ecosystems in their “Manual for Cities.”

If sustainable development practices are to match the pace of rapid changes resulting from urbanisation, the urban knowledge gap must be quickly bridged. Recent literature indicates that urban biodiversity contributes to multiple ES that are very important for the well-being of urban residents. Examples of important UESs include (i) reductions in local air pollution (Gomez-Baggethun et al. 2013); (ii) reductions in the urban heat island effect (Schwarz et al. 2011); (iii) direct health benefits, such as a lower prevalence of early childhood asthma (Lovasi et al. 2008), reduced mortality, and general health enhancements (Maas et al. 2006; Mitchell and Popham 2008), and (iv) enhanced public ecological knowledge and awareness of sustainability challenges. Such UESs are generated by a diverse set of land uses, including parks, cemeteries, golf courses, watercourses, avenues, gardens and yards, verges, commons, green roofs and facades, sports fields, vacant lots, industrial sites, and landfills. Thus, the management of urban ecosystems must be connected to the social–ecological dynamics of developed land. Furthermore, the dependence of cities on surrounding landscape and its biodiversity as well as ongoing interactions between processes occurring in urban, peri-urban, and rural contexts are essential for sustaining the production, enhancement and maintenance of UESs and overall urban resilience.

### Urban Ecosystem Services Versus Ecosystem Functions

ES are the subset of ecological functions (physical, chemical, and biological processes) that are directly relevant or beneficial to human well-being (De Groot et al. 2002). Examples of ecosystem functions include provisioning of wildlife habitat, carbon cycling, decomposition, primary productivity, and nutrient cycling. Urban ecosystems, such as urban wetlands, forests, parks and estuaries, can be characterized by the processes, or functions, that occur within them. The services provided by ecosystems are produced by the functional attributes of ecological communities; in turn, these functions can be characterized by ES indicators and service providing units (SPUs), which are segments of a component of populations, species, functional groups (guilds), food webs, or habitat types that collectively provide the service in a given area (Kremen 2005).

Most of the research on urban biodiversity and ecosystem functioning (BEF) has focused on the role of species richness as a measure of diversity, but ecosystem functioning also depends on the identities, densities, biomasses, and interactions of populations of species within a community and the aggregate abundance and spatial and temporal variation of these attributes. ES and their contribution to quality of life, human health, and well-being are dependent upon the level of biodiversity at the ecosystem and landscape levels. There is still no empirical evidence addressing whether ecosystems need species to deliver more UESs; ecosystems do not necessarily provide more or better UESs when the level of biodiversity is changed. Some studies show that some ecosystems need only a few species to deliver what we want from them. In addition, some systems face a reduction in UES delivery when biodiversity is high due to competition between species. In terms of land use changes, it is important for the resilience of an urban system to maintain high levels of biodiversity from an ES point of view because the higher the level of biodiversity, the higher the resilience, potentially. Set against this background, to manage UESs in the urban context, we need to understand how changes in the community structure affect the magnitude and resilience of ES over space and time (Kremen 2005).

A recent comprehensive quantitative review (Cardinale et al. 2012) examined 20 years of literature on the relationship between BEF outside of the urban context. The authors argue that BEF research should inform the expanding biodiversity and ecosystem services (BES) research for implementation in planning and policymaking contexts, especially in cities. As with most ES, specific services can only be properly integrated with policy and planning after additional research on BEF relationships and links between ecosystem functions and services are understood. Kremen (2005) suggests a framework for linking BEF and BES research that overlaps with the review by Cardinale et al. (2012), who identifies the following: (1) key species or traits providing ecosystem functions, (2) relationships between ecosystem function and community assembly and disassembly processes, (3) environmental factors influencing the production of ecosystem functions, (4) spatio-temporal scales relevant to both SPUs and their functions, and (5) specific relationships between ecosystem functions and ES. The latter can be identified by examining socio-economic and ecological contexts where a given function is directly relevant to humans.

Further, Daily et al. (2009) suggest that the translation of ecosystem conditions and functions into ES requires interdisciplinary and user-oriented research, including (1) collaborating with stakeholders to define services about which people care (e.g., Carpenter et al. 2006; Cowling et al. 2008), (2) developing transparent, flexible models of ecological production functions at scales relevant to decision making, and (3) testing and refining these models in systems around the world to derive general insights (Ricketts et al. 2008). What remains to be explored both theoretically and empirically are the relationships between ecosystem functioning and ES in urban contexts.

### Objectives of the Review

There are a number of comprehensive quantitative and qualitative reviews of global ES (e.g., Seppelt et al. 2011; Cardinale et al. 2012; Hernández-Morcillo et al. 2013), but quantitative comprehensive assessments of ES in an urban context are still rare and very needed at this time. The following questions have not been addressed by the literature: What types of UESs are the focus of current research, and what types of urban land use are examined? Are models used to quantify and assess UES? Are trade-offs and synergies between UES as well as between UESs and other quality of life goals considered? Finally, do studies on UES engage stakeholders in a way that relates to management, policy, or planning practices? This review study seeks to better understand linkages and knowledge of BEF in cities. The statement mentioned earlier that few if any of the reviewed studies examine biodiversity and ES relationships makes clear that BEF in cities is understudied and research is crucial for better understanding links between BEF and UES.

Against this background, we present a comprehensive review of current research on UESs—the first review that focuses exclusively on cities. So doing, first, we describe the materials, methodological design, and quantitative results of the review study. In the discussion, we describe definitions of UES and ecosystem functions and explain their dynamics. We then report which types of UESs are analyzed in the reviewed studies, how provisioning and demand are treated, what methods and indicators are used for analyzing UESs and to what extent implementation and stakeholder engagement are integrated into UES studies.

## Materials and Methods

This UES review is a meta-analysis of published scientific papers. The following search terms and Boolean operators were used for a literature search through the ISI Web of Science to identify studies suitable for inclusion: (i) urban AND ecosystem AND services, (ii) urban AND ecosystems, (iii) urban AND environment, (iv) urban AND land AND use OR cover, (v) urban AND ecosystem AND value OR valuation. These search terms generally cover the topical area of UES.

The search returned 393 unique records. The title of each paper was checked for relevance. Those not focused on the urban context were removed. We also removed studies that were reviews of previous work. As a result, 176 studies were discarded, and 217 articles were included for in-depth analyses. Due to the interdisciplinary and broad character of the subject of “UES,” journals in which these 217 papers were published span over a range of disciplines including geography, ecology, landscape ecology, biology, land use science, planning, forestry, computational science and remote sensing (see Electronic Supplementary Material).

**Fig. 1:**
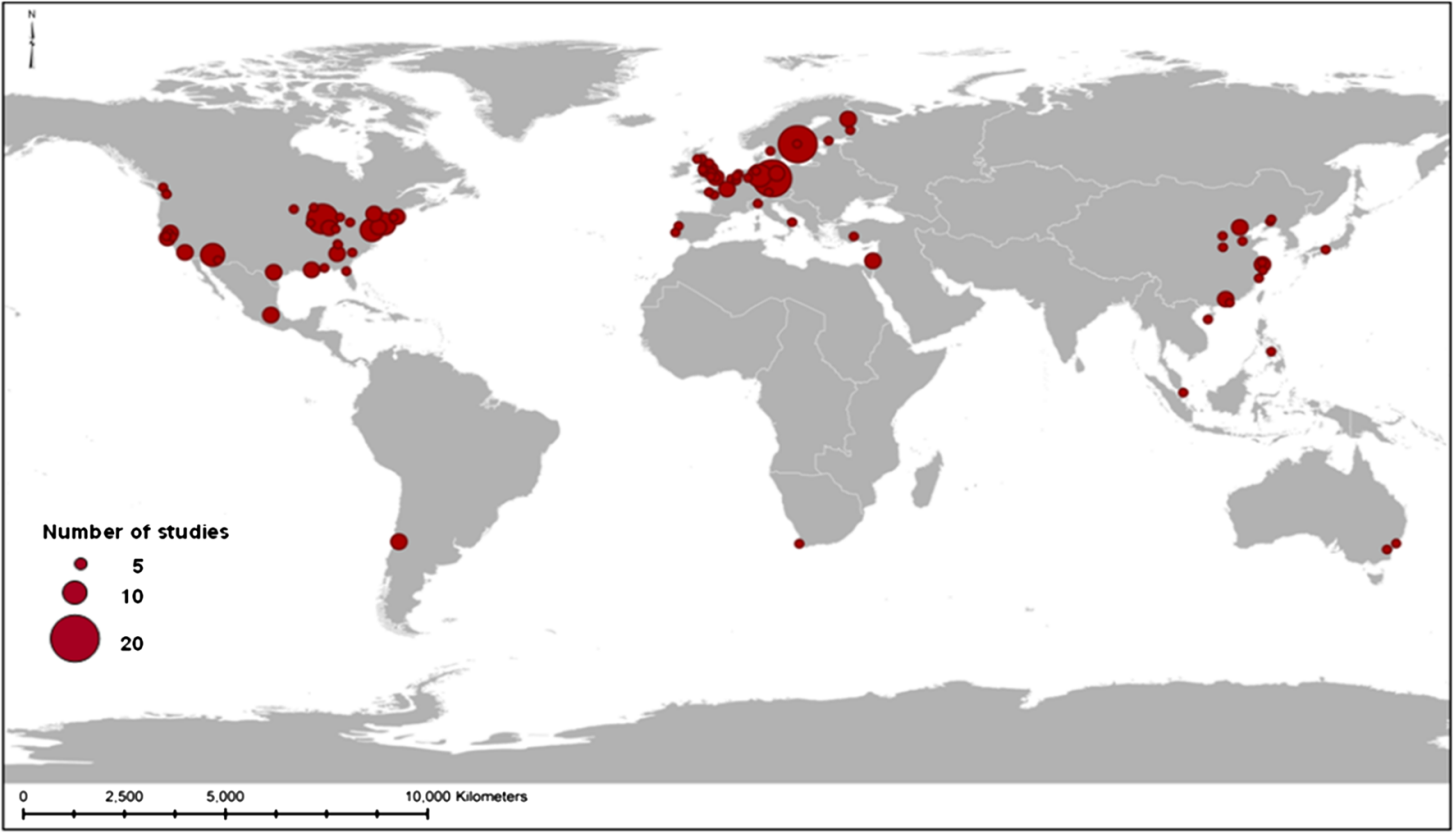
Geographic distribution of 217 UES studies

**Fig. 2:**
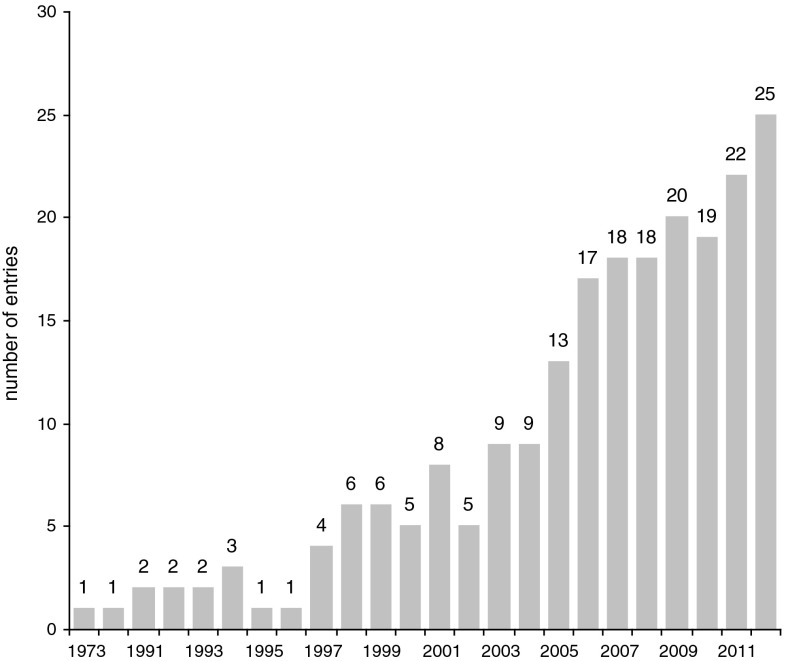
Number of articles published on UES between 1973 and 2012 (*N* = 217)

**Fig. 3:**
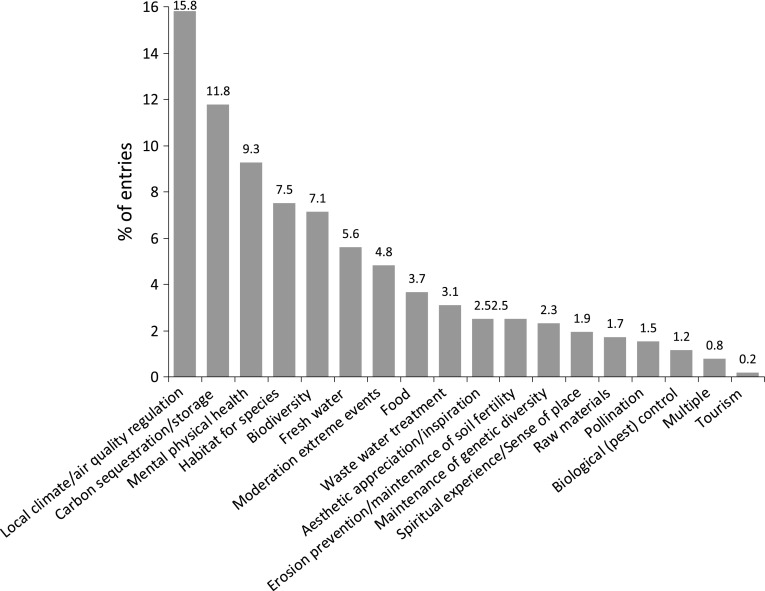
Type of ecosystem services analyzed (% of 217 entries)

**Fig. 4:**
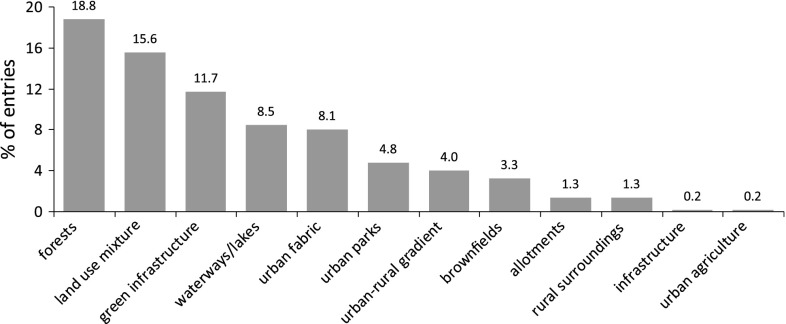
Service providing units analyzed sorted according to the number (% of 217 entries)

**Fig. 5:**
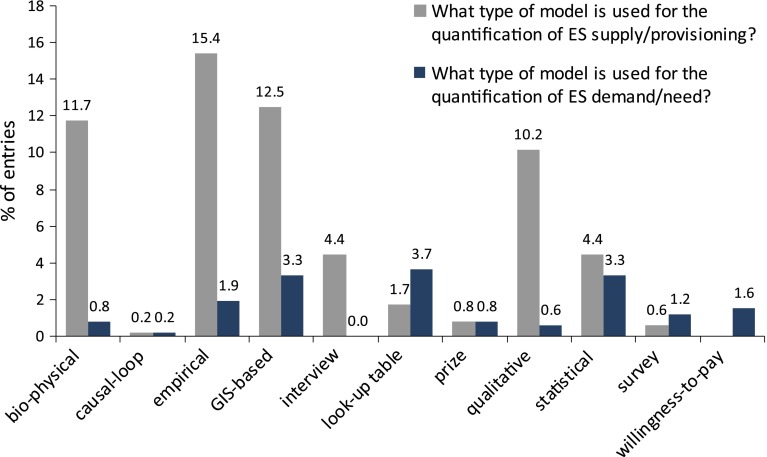
Models used to analyze and assess UES demand and provisioning (% of 217 entries)

**Fig. 6:**
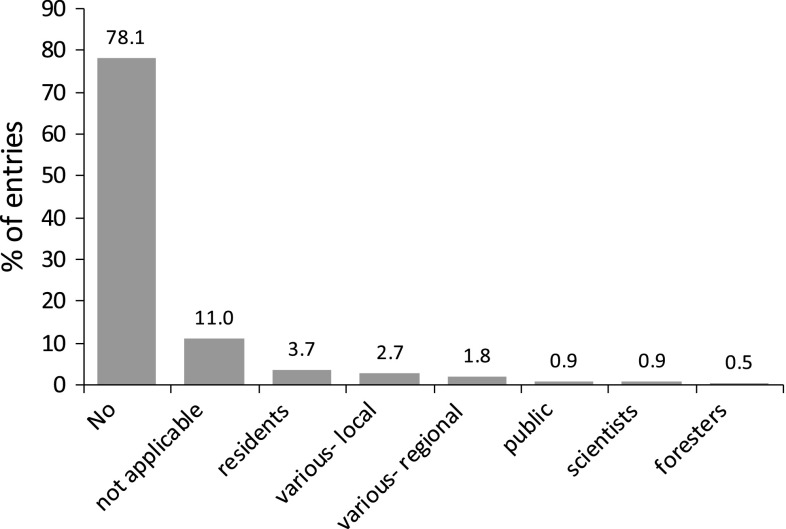
Stakeholders involved in UES analysis and assessment (% of 217 entries)

**Fig. 7:**
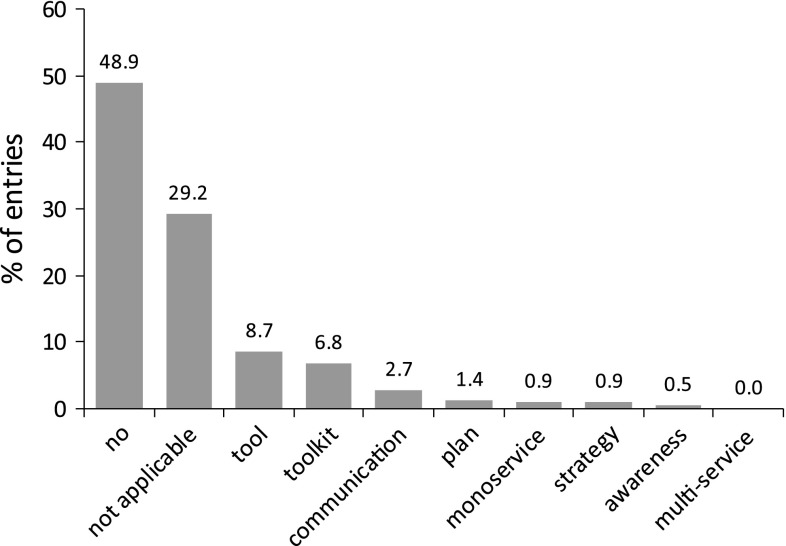
Methods of implementation of UES valuation (% of 217 entries)

Papers were analyzed using a list of assessment criteria (in the form of questions/choices; Table 1), which was developed based on criteria used in existing reviews on ES (Table 1) and issues unique to urban systems, such as different urban scales and planning/implementation issues. The quantitative results of the criteria analysis are shown in Figs. 1–7.

**Table 1:** Criteria for the paper analysis

Criterion (question)	Possible entries
Which type(s) of ES are analyzed?	Provisioning, regulating, supporting and biodiversity, cultural, not applicable
Which number of ES is analyzed?	Numeric answer
In which country is the case study located?	Name of the country where the study is located
In which city (region) is the case study located?	Name of the city where the study is located
Does the paper explicitly mention “urban ecosystem services”? Is a specific vulnerability to change (climate change, loss of BD, etc.) considered? Are off-site effects considered? Is a model used for the quantification of ES provisioning? Is a model used for the quantification of ES demand? Are synergies considered?	Yes, no, not applicable
What is/are the specific ES analyzed?	Food, raw materials, fresh water, medicinal resources, local climate and air quality regulation, carbon sequestration and storage, moderation of extreme events, waste water treatment, erosion prevention and maintenance of soil fertility, pollination, biological (pest) control, habitat for species, maintenance of genetic diversity, biodiversity, recreational and mental and physical health, tourism, esthetic appreciation and inspiration for culture, art and design, spiritual experience and sense of place, other, not applicable
Which indicator(s) are used?	Indicator and unit (e.g., carbon storage in MgCO_3_)
Does the paper deal with ES potential or demand and provisioning?	Potential, demand and provision, demand, not applicable
What scale is used?	City region, city, neighborhood, site, not applicable
Which SPUs is the paper dealing with?	Forests, urban agriculture, urban parks, waterways/lakes, cemeteries, urban fabric, allotments, rural surroundings, infrastructure, brownfields, land use mixture, urban–rural gradient, green infrastructure, other, not applicable
What is the temporal scale?	One time step, time series analysis, not applicable
What is the relation between demand and provisioning?	Local, regional, distal (teleconnections), not applicable
What kind of valuation methods/indicators is applied?	Monetary, non-monetary, both, not applicable
What type of model is used for the quantification of ES supply/provisioning? What type of model is used for the quantification of ES demand?	Bio-physical, GIS-based, statistical, qualitative, causal loop, look-up table, willingness-to-pay, survey, interview, conjoint analysis, prize, trading, REDD, risk assessment, empirical, other, not applicable
Are trade-offs considered?	No, between ES, between land use and ES, between ES and quality of life, between ES and economy, other, not applicable
Are stakeholders involved within the assessment?	Policy makers, policy analysts, NGOs, land owner/lords, scientists, firms/industry, farmers, foresters, public, residents, tourists, various, various-local, various-regional, EU-policy makers, no, not applicable
Is the approach implemented?	Tool, toolkit, monoservice, multi-service, test phase, plan, strategy, communication, awareness, no, not applicable

## Results

Most of the existing studies on UESs were undertaken in Europe, North America, and China (Fig. 1) with an increasing number of papers from 1975 onward (Fig. 2). With the exception of China, nearly all the empirical evidence about the provision of and demand for urban ecosystem goods and services and the implementation of study findings into land use policy has been gathered in the Western developed world.

Almost 50 % of the ES assessed within the reviewed papers are regulating ES. Twenty percent of all the analyzed services are supporting services, 15 % are cultural services and 11 % are provisioning services. Few studies discuss the relationship between urban biodiversity and ES such as, e.g., Bezák and Lyytimäki (2011). Also rare are studies investigating urban soil supporting services such as by Haase (2009) on groundwater recharge and run-off generation. Figure 3 shows which types of UESs were analyzed in the reviewed studies. Most of the investigated regulating services focus on local climate, air quality regulation, and carbon sequestration and storage. By contrast, biological regulating services, such as biological pest control, are analyzed in only 1 % of all the reviewed papers. In terms of the number of UES valued within each reviewed paper, the results of the review show that the numbers reveal a lack of multi-service valuations; almost 60 % of the reviewed studies focus on a single UES.

Figure 4 shows the variety of ecosystem service providing units (SPUs) examined in the reviewed papers. Most of the studies analyze ESs that are generated by forest areas or patches (18.9 %), land use mixtures (15.6 %) or urban green infrastructure (parks, leisure areas; 11.7 %). Furthermore, Fig. 4 highlights the need to consider industrial and brownfield land uses, which are gaining importance, particularly in stagnating and shrinking cities, as well as allotment and community gardens, which form an important niche of food supply for an increasing number of cities (Barthel et al. 2010).

## Discussion

### The Temporal Dimension: Dynamics of UES

An analysis of the relationships between processes of urbanisation, including impact assessments of plans or projects, and the flow of ES is essential to support informed decision making. Information is needed both to assess the consequences of past urbanisation trends and planning decisions and to inform the possible future impacts of pathways of urbanisation and planning decisions.

Thematically, the reviewed papers cover a diverse range of studies. Most papers presented information on the change of UESs at a regional or city scale, whereas only five papers presented results from more fine-grained analysis at a neighborhood or site scale. Approximately half of the studies provided some kind of historic analysis. Only two studies undertook long-term assessments (Imhoff et al. 2004; Haase 2009). For instance, Haase (2009) analyzed the changes of regulating ES related to hydrology for the city of Leipzig over a period of 130 years. Another study established the impacts of urbanisation on net primary productivity (NPP) for the coterminous United States by contrasting pre-industrial urbanisation with the state in 1995 (Imhoff et al. 2004). All other papers included in this review covered shorter periods of 20–40 years. Other studies projected the accumulated benefits of existing and/or additional urban trees and forests over certain time periods (McPherson 1992; McPherson and Rowntree 1993; McPherson et al. 1997; McPherson and Simpson 2003; Morani et al. 2011). The study by Schetke and Haase (2008) was the only one found to combine historic analysis with a scenario approach.

Overall, the studies encompassed a broad range of ES, though regulating services, such as carbon sequestration and storage, the regulation of air temperatures, air pollution removal, and/or storm water runoff, were the most common. A small number of studies dealt with provisioning services (food, raw materials), and only one study examined the effects of urbanisation on a supporting service—the pollination of plants by bumble bees (Jansson and Polasky 2010). Only three of the selected papers, two of which referred to the same study, dealt with a change in cultural ES, such as recreation (Schetke and Haase 2008; Schetke et al. 2010).

A few studies covered more than one dimension of ES, i.e., studied concurrent changes of services, such as the provision of food, the regulation of microclimates and storm water retention. Among these are several studies assessing the multiple regulating ES of urban forests. For instance, Morani et al. (2011) assessed the potential impact of New York’s MillionTrees NYC program on carbon storage and air quality improvement over a period of 100 years. However, none of the studies included in this review addressed trade-offs or synergies between the various UESs.

The majority of the reviewed studies used a spatial approach. These typically related land use and/or land cover change derived from maps, aerial photographs and satellite imagery to ES. The results were presented at a variety of different levels of resolution. Studies such as Burkhard et al. (2009) and Kroll et al. (2011) provided ES assessments in the form of land cover maps for land cover/land use types. In other studies, information was provided at the aggregate level of administrative subunits (Escobedo and Nowak 2009) or for the city level (McPherson et al. 1994).

Even when land use remains the same over time, its character may change. For instance, urban green spaces and agricultural land may be managed more or less intensely, and trees may mature or be removed. The studies that used land use/land cover data rarely addressed these dynamics. For instance, in Haase’s (2009) assessment of long-term hydrological changes for the city of Leipzig, the average values of surface cover remained the same for land use types over a period of 130 years, whereas the percentage cover of the land use types changed. By contrast, Burkhard et al. (2009, 2011) were able to show that the increase in the productivity of farming in the Leipzig region overcompensated for the loss of farmland due to urbanisation.

### The Facts: Indicators for UES Assessment

Understanding the factors influencing UESs requires the use of linked or bundled indicators that track driving social–ecological forces as well as pressures on ecosystems. Researchers are increasingly developing and testing ES indicators from a wide scale to a local site scale. Indicators allow researchers to analyze, monitor, and efficiently measure the conditions, characteristics, trends, and rates of change of UESs (Layke 2009; Sparks et al. 2011) and help reduce complexity. An indicator is defined as a measure or metric based on verifiable data that conveys information about more than itself. For example, the size, structure, and accessibility of urban green areas and the number of visits per day are indicators for recreational UESs produced by city green areas. Indicators help track and communicate how ecosystems support the physical, economic, and socio-cultural well-being of people. With the help of indicators, the complexity can be condensed to a manageable level that can inform decisions and actions (Bossel 1999). Best case scenario, public and private sector decision makers can base decisions on scientific evidence, identify and prioritise measures, track progress toward targets, and effectively communicate the value of UESs (Layke 2009).

The approaches to analyzing and assessing UESs are relatively new and still evolving. There are numerous UES indicators and metrics with differing quality and applicability in use, including many of which are still conceptual in nature and lack demonstrated relevance. In general, the most common and developed indicators are for provisioning UES which is most likely due to data availability (Sparks et al. 2011). However, the conceptual and data underpinnings for indicators remain underdeveloped (Millennium ecosystem assessment 2005a; Boyd and Banzhaft 2007; Wallace 2007; Turner et al. 2008; Layke 2009; De Groot et al. 2010a, b; Sparks et al. 2011). The choice of services to assess and indicators to use in assessments is often determined by policy objectives and data availability. Indicators have to be adequate for the particular service, comparable and simple enough to be intuited and easily communicated (Sparks et al. 2011). The indicators applied by the studies we analyzed were developed for a variety of purposes (e.g., indicators from narrower environmental fields, economics, agriculture, or tourism); therefore, they neither focused on the contributions of UESs to human well-being nor helped public/private sector decision makers integrate UESs (Layke 2009).

The quality and quantity of data vary widely from scale to scale. Available data are not always sufficient to support the use of particular indicators. Applying the ES framework requires information at multiple spatial and temporal scales; therefore, monitoring systems need to gather data with sufficient regularity and at a relevant scale to track changes at a rate appropriate to the “characteristic scale” of ecosystem processes and flows of service (Millennium Ecosystem Assessment 2005a).

Despite the growing literature on UES indicators, there are still many challenges to the development of indicators. Redundancy and double counting are as much issues in indicator development as they are in UES assessment. The number of indicators that convey very similar information under different names needs to be reduced. In addition, how indicators are linked to services and benefits remains an important and unresolved issue. An indicator’s capacity to convey the characteristics of a UES at multiple spatial and temporal scales varies widely between services. Only sensitive indicators are able to detect changes in time for prompt policy adjustments. Some researchers claim that one indicator covers a number of issues related to a particular UES, whereas others use several indicators focusing on only one aspect. However, in general, a single indicator is not sufficient for most assessment purposes. In addition, the indicators used in studies we reviewed were inadequate to characterize the diversity, quality and complexity of the benefits people derive from ecosystems. As with all models, indicators are intended to reduce complexity and therefore do not provide a complete picture of all services or indeed even a particular service (Layke 2009). Indicators often mix structural or compositional attributes with functional ones; structure and composition are easier to measure than function.

An important distinction can be made between different types of indicator metrics, including supply, consumption, and sustainability (Sparks et al. 2011). Indicators of biodiversity or the stock of particular components imply something about the ecosystem’s ability to provide ESs, but say little about the benefits people effectively derive from those services. Similarly, consumption indicators provide information about the flow of benefits but say little about the sustainability of these benefit flows. De Groot et al. (2010b) distinguish two main types of indicators: state indicators, which describe what ecosystem component or process is providing the service (e.g., the number/area of landscape features with stated recreational value), and performance indicators, which describe how much of the service can potentially be used in a sustainable way (the maximum sustainable number of people and facilities).

Communicating about ES in a comprehensive way is a challenge regardless of whether the end user is a planner, policy maker, manager, or practitioner. ES indicators have to communicate ES characteristics clearly without ambiguity, avoiding differing interpretations of the state or trend of the ES. In addition, indicators have to be easily understood by policy makers and other non-scientific audiences so that the importance of UESs for citizens’ economic, physical, or spiritual well-being is well understood.

### Two Sides of a Coin: Demand and Provisioning of Urban Ecosystem Services

Ecosystems deliver several services at the same time, potentially create synergies and trade-offs among UES and between these services and other factors. The equitable distribution of resources and social–cultural demand for ES are rarely simultaneously evaluated, yet it is clearly important to not only identify services provided by urban ecosystem but also understand social–cultural needs for services and identify locations where needs are unmet. Additionally, there can be ambiguity in the way different researchers distinguish between services, functions, and benefits and therefore valuation discrepancies arise.

Useful classification and evaluation schemes of ES demand need to take into account the complex nature of ecological systems, including their nonlinear nature, the joint production of ESs, multiple spatial–temporal scales, the variety of beneficiaries, and decision contexts in which ES are evaluated (Fisher et al. 2009). Developing methods that are able to account for these multiple perspectives is one of the pervasive challenges in making ecosystem approaches to urban planning operational at the policy and decision making levels.

Regulating services play a major role contributing to human well-being in cities; they can help reduce urban heat island effects and mitigate climate change and air pollution. Whereas well-proven indicators and empirical studies exist for regulating services, basic knowledge gaps still need to be closed for cultural and provisioning services. Approximately 15 % of all reviewed studies use value indicators for local climate and air quality (33) regulation and/or carbon sequestration and storage (32). It is interesting that carbon sequestration and storage make up such a large component of ES assessment given the recent criticism of their utility in urban contexts (Pataki et al. 2011). Additional studies of provisioning services include those dealing with energy supply (Kroll et al. 2011; Lundy and Wade 2011). Almost 7 % of the assessed papers address biodiversity valuation (37) and 7.5 % address habitats for species (39). Indicators for recreation and mental and physical health (48), habitats for species (39), and biodiversity (37) were assessed more than 30 times each. Indicators for biological pest control (6), tourism (1), and medicinal resources (0) were addressed less than ten times. All other UESs described in TEEB were assessed in 10–30 of the studies reviewed. The remaining studies (33) primarily address ESs in the regulating category, including wetland analysis (Barthel et al. 2005), indicators for nutrient removal (Tong et al. 2007), and yield stability studies (Schetke et al. 2012). Cultural service studies used indicators for educational value (Lundy and Wade 2011) and communication.

A broad diversity of indicators has been used to assess UESs in the reviewed studies, and most indicators were only used once. Local climate regulation, fresh water supply, and recreation were the three most frequently investigated UESs. In 37 papers dealing with local climate regulation, more than 20 different indicators were used. In 24 papers, more than 15 different indicators were used to measure carbon sequestration. A large number of different indicators were used as biodiversity measures, including the number of species and bird or butterfly diversity. Cultural service indicators and metrics included access, the distance to green space, the number of visitors, willingness-to-pay, human health, opportunities in recreation, the motivation of users, the numbers of features with specific value, money flow, and increases in real estate value. Esthetic appreciation, inspiration for culture, spiritual experience, and regional identity were rarely considered. Most indicators were derived from data on the structure (extent/condition/stock) of underlying elements of an ecosystem or on the provisioning or use of services by humans. However, there were few assessments of the sustainability of UESs.

### The Economic Dimension: Monetary and Non-monetary Valuation

The pluralism of values with respect to UESs has been highlighted from both theoretical and empirical perspectives (Chiesura 2004; Hubacek and Kronenberg 2013). Thus, different values and perceptions should be considered to make well-informed decisions in the management of urban ecosystems (De Groot et al. 2010b). The choice of which specific values should be assessed and articulated in the processes of urban planning depends on the characteristics of the UESs that are being valued and the institutional and socio-cultural contexts in which decisions take place. Of the reviewed studies, 156 applied exclusively non-monetary indicators and methods to assess UES values, while 77 studies used both monetary methods and non-monetary indicators and methods.

Although there has been a recent thrust to apply monetary means to value ES and biodiversity, these means can be inappropriate when they fail to take into account the totality and plurality of values, which are also characteristic of non-monetary indicators (TEEB 2011). Within the group of non-monetary valuation methods, a broad number of methods, criteria and indicators have been developed to assess UESs, which can be broadly divided into ecological and socio-cultural methods.

Ecological valuation does not directly consider human needs or stated preferences and wants. It instead considers physical or nonphysical environmental outputs, which have indirect value for humans (Winkler 2006). The non-monetary assessments using ecological indicators and criteria that we reviewed focused on regulating and supporting services. Among regulating services, air purification (using, e.g., the leaf area of trees and shrubs as preferred indicator; Escobedo and Nowak 2009; Jim and Chen 2009; Escobedo et al. 2011), the cooling effect of trees and parks (e.g., Upmanis and Chen 1999; Shashua-Bar and Hoffman 2000) and carbon storage and sequestration (Lal 2004) are of primary interest. Wastewater treatment, pollination and the moderation of extreme events (Costanza et al. 2012) are less frequently considered and should be more strongly integrated into future research. Chapin et al. (2000) highlighted the importance of habitat and species diversity for the functioning of ecosystems and the support of ES (Clergeau et al. 1998; Zerbe et al. 2003). By contrast, genetic diversity (Dobbs et al. 2011) and medicinal resources were less frequently examined.

Freshwater is a vital good and therefore most often investigated within provisioning services (followed by food production), using such indicators as groundwater recharge (Haase 2009), the relation of demand and provisioning (Fitzhugh and Richter 2004), and evapotranspiration (Schetke and Haase 2008). Because these ecological indicators reveal environmental outputs and functioning as well as human well-being (Millennium Ecosystem Assessment 2005a), research linking ecological and cultural services via an interdisciplinary approach is crucial (McMichael 2008).

Methods for assessing socio-cultural indicators and values take into account socio-cultural perceptions of ES in terms of their importance to human well-being. They are mainly used for ES that are not valued within markets (Chan et al. 2012 for a theoretical explanation; Ambrey and Fleming 2011; Calvet-Mir et al. 2012 for case studies). As one can see from Fig. 5, surveys and other qualitative means to elucidate socio-cultural values were found in far less studies than those using biophysical and monetary methods to capture ES values. Qualitative and quantitative social research such as questionnaires, focus groups and interviews capturing non-monetary values, were most often used with cultural ES (Chiesura 2004; Maas et al. 2006; Mäkinen and Tyrväinen 2008), a result that is not surprising considering the highly subjective, intangible and incommensurable nature of cultural services (Chan et al. 2012). Nevertheless, some studies used socio-cultural methods to assess other ES. In some cases, this assessment was performed to attain information on the complexity of land management and implications for the provision of ES (Barthel et al. 2005) or assess concepts of land managers (Niemelä et al. 2010). Others used a combination of socio-cultural and ecological methods to assess the effects of land management practices on regulating or supporting services (Florgård 2000) or to compare ES with the perception of well-being and recreational opportunity (Fuller et al. 2007; Rall and Haase 2011). Although socio-cultural methods and indicators are important for obtaining stakeholder values, they are also time-intensive and costly. It remains to be seen whether more sophisticated socio-cultural measures that account for the complexity of multiple perspectives while incorporating UES trade-offs can be developed.

Approaches to economic valuation have the common characteristic of using monetary units as an indicator. Nevertheless, this indicator can be derived by different methods. Provisioning UESs, consisting of directly marketable goods, such as drinking water, food, and raw materials, are directly valued through market observations of reference prices (Tong et al. 2007). By contrast, studies that examined regulating UESs used revealed preference methods to derive UES values based on secondary markets. Among the monetary approaches used in the reviewed studies, revealed preference was the most common. These studies evaluated indicators such as the replacement cost of seed dispersal (Hougner et al. 2005) and the abatement cost of air pollution (Jim and Chen 2008). The UFORE/iTREE model (Nowak and Crane 1998), which was applied in various studies (McPherson et al. 1999a, b; Soares et al. 2011), also determines the monetary values of regulating UESs by urban forests via revealed preference approaches. Due to the data requirements of this approach, UFORE/iTREE is usually applied to a single UES or several closely related UESs.

Hedonic pricing methods are often used to determine the value of cultural UESs, such as the esthetic of green areas (Tyrväinen 2001). They derive the monetary values of particular ecosystem characteristics from comparisons of market prices (Boyer and Polasky 2004) that usually rely on real estate markets. A major difficulty in the application of hedonic methods is the limitation to the assessment of use values, such as those provided by cultural services and some regulating services, depending on the scale. Hedonic methods require large data sets and complex methods of data analysis, e.g., regression analysis. Another monetary valuation approach is contingent valuation (Boyd and Banzhaft 2007; Tong et al. 2007), which does not rely on existing markets. It uses stated preferences collected through surveys. This approach is, in that aspect, closely related to socio-cultural valuation methods. To obtain socio-cultural values, methods are needed that often demand the use of holistic approaches that may include qualitative measures, constructed scales, and narration (Patton 2001; Chan et al. 2012). In some cases, translating these values into quantitative metrics is difficult or senseless. However, scientists have developed toolsets to measure values such as sense of place (Williams and Roggenbuck 1989; Shamai 1991) and traditional ecological knowledge (Gomez-Baggethun et al. 2010) using constructed scales when appropriate. Additional sets of values that can be labeled as socio-cultural include sense of community, social cohesion, and spiritual values (Gomez-Baggethun et al. 2013). Contingent valuation allows for simultaneous accounting of multiple ES. However, in a complex policy setting involving multi-dimensional scenarios, respondents may not be able to accurately state their preferences (Nijkamp et al. 2008). Although temporal and spatial value transfers are often conducted (Kreuter et al. 2001; Zhao et al. 2004; Troy and Wilson 2006), monetary values are generally highly context dependent (Mäler et al. 2008) with regard to socio-ecology, politics, and economics at any given time. Monetary valuation approaches can provide relevant information for policy decisions affecting ecosystems and the services they provide (Costanza et al. 1997). However, in practice, their focus tends to be too narrow to encompass the total complexity of socio-ecological systems (Chee 2004). The integrated assessment (Brouwer and Van Ek 2004) of monetary values in an urban context is strongly needed.

Our review finds that a broad number of different criteria, indicators and methods have been used to determine UES values whereas a large gap between the approaches and the underlying disciplines lies in the coherent definition of UES, functions, benefits, and values. Links between these concepts are only established for economic methods (De Groot et al. 2002), and a similar approach is lacking for non-economic values. Because many studies focus on a single or limited number of UESs, the existing research is unable to account for value pluralism and UES trade-offs. Integrated valuation methods, such as multi-criteria analysis and institutions through which integrated values can be articulated, are sorely needed to make UES valuation applicable for local and regional planners (Brouwer and Van Ek 2004; Rodríguez et al. 2006). Further, evaluations of the use and implementations of the concept of UESs by urban planning authorities in different geographic and political contexts would also be helpful (Niemelä et al. 2010). Finally, there is a need for comparative testing of applied methods, which is often insufficiently reported or completely absent in the literature.

### Data and Models of UES Quantification

Quantitative modeling plays a major role in assessing UESs. Because the urban ecological system is very different from non-urban ecological systems (Gomez-Baggethun et al. 2013), models used for urban valuation need to be adjusted to the complex, multi-functional urban environment (Pataki et al. 2011). Various models are used to value ES demand and provisioning, including biophysical, empirical, GIS-based, statistical and survey-based models and less widely applied approaches such as qualitative studies, causal loops and look-up tables (Fig. 5). In addition, monetary modeling approaches use the identification and valuation of ES as input to cost-benefit analyses (CBA) or willingness-to-pay (WTP) analyses. The quantitative review shows that modeling approaches often value the provision of provisioning ES (provisioning of 368 ES were modeled) rather than demand (demand for 113 ES were modeled). Overall, the supply side has been investigated more often than the demand side. Provisioning studies use empirical (80), GIS-based, (65), bio-physical (61) or statistical (53) approaches, whereas demand is modeled through look-up tables (19) and statistical (17), GIS-based (17) and other (24) approaches.

Bio-physical evaluation models are able to analyze complex ecological systems and impacts but are limited in that they tend to focus on provisioning services. With respect to indicators and service providing units, these models tend to focus on the potential for forests to reduce air pollution (Jim and Chen 2009). One paper used a causal loop method to model demand and the provisioning of ES studying wetland biodiversity responses to land use changes (Eppink et al. 2004).

A large number of studies use empirical methods or models to quantify the provision of ES (70). Most of these analyze the potential for urban green infrastructure to provide regulating services such as air pollution and local climate regulation (23 out of 70). A number of empirical studies examine the provision of biodiversity (9) and carbon sequestration and storage by trees (11). Some empirical studies use a combination of quantitative and qualitative assessment data, utilizing land cover data and GIS (Burkhard et al. 2009, 2011).

GIS-based models have been used to assess and analyze the provision of UES and, to a lesser degree, have also assessed or analyzed the demand for these services (26). GIS-based models are useful for demand and provision analyses because spatial data, such as land cover and land use data, can serve as a basis for estimating quantities of the particular UESs associated with vegetation types, soil and other landscape features. Moreover, spatial dynamics can reveal heterogeneity and trends in the distribution of UESs over urban landscapes, which can be of importance for urban sustainability planning. Other studies have quantified spatial variation in UES values using a hedonic price model and analyzed spatial relations among biodiversity features to assess habitat supply (Angold et al. 2006).

Look-up tables were generally used to transfer results from previous studies to current studies of interest. Some studies derived monetary values (US$) for specific land use categories (Kreuter et al. 2001) or applied urban tree benefits, such as carbon sequestration and air quality regulation (Brack 2002). Others utilized online mapping tools such as i-Tree (Abd-Elrahman et al. 2010), developed toolkits (Alberti 1999; Troy and Wilson 2006), performed cost-benefit analyses (McPherson 1992; McPherson et al. 1997, 1999a, b) and conducted aerial and satellite photograph analyses (McPherson et al. 2003; Zhao et al. 2004). Studies have applied economic valuation (prices) to existing provisioning of ESs and, to a lesser degree, the demand for ESs. Regression models are often used to analyze and calculate UESs to value provisioning and demand. Most of the reviewed articles examine the city and urban region scales, whereas the neighborhood scale seems to be underrepresented, with studies performed in Shanghai (Yin et al. 2011) and Chicago (Coley et al. 1997).

Using qualitative techniques, studies have explored links between UESs, human behavior and values. Studies using qualitative analysis and survey instruments designed to understand both how human behavior affects the provision of UESs and how people respond to and value ESs allow for a deepened understanding of the linkages between social and ecological dynamics in an urban context. Surveys are often conducted to analyze the recreational potential of urban green areas. They include quantitative questionnaire surveys, which can examine the use, perception (Mäkinen and Tyrväinen 2008; Chiesura 2004) and possible health impacts (Maas et al. 2006) of urban parks. Willingness-to-pay analyses based on survey data are also used to determine the demand side of ecological valuations. For instance, willingness-to-pay analysis was applied to urban forestry (Abd-Elrahman et al. 2010) and an urban gardening project (Barthel et al. 2010).

This review demonstrates that a large number of indicators and models are used for the assessment of UESs, but “[…]practical applications, appropriate methods for identification and quantification of individual services, suitable models, indicators and the integration of system components are still needed” (Burkhard et al. 2010). Most case studies valued ES without detecting temporal changes. In addition, approaches focusing across cities or neighborhoods are almost missing. One way to better explore and use locally existing data for UES assessment is “Virtual Globes”—that is “… technologies offering capabilities to annotate, edit and publish geographic information to a world-wide audience and to visualise information provided by the public and private sectors, as well as by citizens who volunteer new data” (Blaschke et al. 2012, p. 373).

### The Practical Dimension: Implementation of UES

Research is crucial to gain knowledge on ES and to develop approaches for their management. However, the findings need to be effectively transferred from the scientific sphere into policy making to mitigate biodiversity loss and ecosystem degradation. Of the 217 studies examined in this quantitative review, we found 48 studies that address implementation in urban policy making and planning (Fig. 6). Implementation included awareness raising and communication, strategic planning, and the development of tools and toolkits.

Even though the awareness raising and communication of research may be considered a basic step toward implementation, the overwhelming majority of articles included only short, general recommendations for stakeholders, if at all. Only nine studies included more detailed recommendations (i.e., longer than one paragraph). Of these, a few delivered highly technical recommendations (e.g., to optimize vegetative plantings for carbon sequestration; Jo and McPherson 1995) or suggested engineering solutions for freshwater provision and flood mitigation. More often, recommendations were directed generally at land management (e.g., strategies for more efficient nature preservation; Breuste 2004) but without specifying relevant stakeholders, plans and policies. Although general recommendations for land management and planning can be applicable at multiple scales, they are unlikely to help foster change if the results are not communicated directly to stakeholders. Stakeholder communication was found in only nine studies, six of which were not linked to the development of strategies, plans or tools but rather served to exchange relevant information used in model development (McPherson 1998; Mcpherson and Simpson 2003) or to obtain contextual information about land management practices (Barthel et al. 2005; Andersson et al. 2007). Only in three cases was it stated that the results of the study were directly communicated to stakeholders (McPherson et al. 2003; Li et al. 2005; Schetke et al. 2012). However, with the exception of a review of a manuscript draft in one case (Mcpherson and Simpson 2003), the exact form of communication was left unmentioned.

Looking for plans, strategies, frameworks or guidelines to integrate ES in planning and policymaking, we found that, in most studies, links between research and planning were quite limited. Where statements regarding implementation were made, they did not provide detailed reasoning about how and under what circumstances the approach could be implemented. However, some papers highlighted the importance of refinement or adjustment of the approach, their limitations and the complementary measures needed for successful implementation (Hong et al. 2009; Dobbs et al. 2011). Two strands of studies addressing planning and strategies were examined: (i) the development of new approaches for the assessment of UESs for planning or the improvement of existing approaches by incorporating the concept of UESs (Cook 2002) or underlying ecological processes (Lundberg et al. 2008) and (ii) research that generated new knowledge for enhanced recognition of UESs in planning processes (McPherson et al. 1994; Dixon et al. 2006; Paoletti 2009). The two studies that were classified as having a high degree of implementation were both involved in planning processes (Li et al. 2005; Nuissl et al. 2009). The study by Li et al. (2005) was initiated by the Beijing Municipal Institute of City Planning and Design, and the results were also discussed with planners and government officials. In the study by Nuissl et al. (2009), the resulting assessment of the effects of land use changes on landscape functions was used in the city planning process. Not surprisingly, these studies targeted city authorities, who are usually the main actors in strategy development and land use planning. Indeed, most of the studies in which plans or strategies were developed took place at the city level. Those operating at larger scales often addressed multiple UESs.

Tools and toolkits for measuring and assessing ES were designed to support decision making and policy development. Most of these tools and toolkits were developed and tested by scientists without the involvement of stakeholders. Some authors state that specific tools, such as CITYgreen (http://ebmtoolsdatabase.org/tool/citygreen), have been used in practice but do not describe whether the tools were developed in cooperation with stakeholders.

Only three studies involving tools included a level of implementation which was designed to support planning practitioners at site scale or for specific projects. In an integrated socio-ecological impact assessment of alternative flood control policies in the Netherlands, Brouwer and van Ek (2004) provided a cost-benefit analysis and multi-criteria assessment as part of a decision support system for planning. The results were discussed with an administrative steering group. Multiple UESs were included in a sustainability assessment tool for planning the interim use of brownfields (Rall and Haase 2011), where stakeholder interviews are used to inform study results and produce recommendations for the application of the tool. The highest level of implementation was found in Schetke et al. (2012), who developed a multi-criteria assessment and decision support system to evaluate the sustainability, resource efficiency and recreational benefits of the development of greenfield and infill sites. The development of the tool was stakeholder driven, involving planners and decision makers in the selection, weighting and testing of indicators.

Although the concept of ES is nascent and basic research is still needed, a surprisingly large proportion of the studies reviewed included little or no information on implementation. More research is needed to better address the question of whether implementation was simply not elaborated in the papers or not included in the study design. There may have been more communication with stakeholders during the research, but the authors of the reviewed papers gave the issue of communication little attention. Therefore, we conclude that the general level of implementation is low. If the results of ES research are to influence the appreciation and management of ES in urban areas, the transfer of knowledge and methods gained from ES research into planning and policy making needs to be improved (Seppelt et al. 2011), which means not only developing strategies and tools that can be understood, accepted and applied by stakeholders but also effectively communicating the results to specific user groups and considering when and if to actively involve stakeholders in the development process. Some models and tools used in the research may be too complex for use by stakeholders. However, their basic assumptions and limitations can still be shared with relevant stakeholders along with the results. In other cases, scientifically derived models and tools can be used or may even be explicitly designed for use by stakeholders. Here, exchanges between scientists and stakeholders in the development process can create new insights and enhance the usability, transparency and acceptance of tools and models.

### The Participatory Dimension: Stakeholder Involvement

Stakeholder involvement is generally recognized as being a fundamental element of the ES research agenda (De Groot et al. 2010b; Seppelt et al. 2011; Daniel et al. 2012). Although the involvement of stakeholders within environmental research is not without caveats—for example, the reservations of planners to work with models and uncertainties and arrogance of science against practitioners—and requires careful planning (Seppelt et al. 2011), it has the potential to illuminate understanding of land use impacts, trade-offs and possible management options and pave the way for more effective decision making (Millennium Ecosystem Assessment 2005a).

Despite the high level of importance of stakeholder involvement discussed in the general literature, only 24 of the 217 studies (11 %) under review involved stakeholders (e.g., planners, forest managers, farmers, land owners). In terms of scale, the overwhelming majority of studies involving stakeholders were focused at the local and regional levels. Approximately half of the studies concentrated on cultural services, whereas few focused on provisioning and supporting services (Fig. 7).

Three purposes of stakeholder involvement were detected in terms of the nature of involvement: (1) determining the understanding and planning relevance of the concept of ES, (2) the development of a framework and selection of relevant ES and indicators, and (3) data collection and the assessment of ES. The majority of papers used stakeholders to assess ES through surveys, workshops or interviews. A few studies concentrated on the effects or costs of management decisions for assessing regulating (Escobedo et al. 2008), supporting (Florgård 2000), or multiple types of ES (Barthel et al. 2005; Rall and Haase 2011; Schetke et al. 2012). Most examined cultural ES exclusively (Kliskey 2000; Chiesura 2004; Fuller et al. 2007; Mäkinen and Tyrväinen 2008) where information was obtained from city residents or users of various urban green spaces about such aspects as motivations for use, perceptions, values, and physical and psychological well-being. This result mirrors the findings from others (Daniel et al. 2012) that qualitative methods are the primary means used to assess cultural ES.

This review revealed a number of gaps related to stakeholder involvement, particularly in three key areas. Countries outside of the EU and US were underrepresented in terms of stakeholder involvement, but even within the EU and US studies, stakeholder involvement was mostly limited to one scale or one type of stakeholder. This finding stands in contrast to recommendations from TEEB (2011) and others (De Groot et al. 2010b; Müller et al. 2010) who argue that to adequately analyze the effects and trade-offs of land use decisions, all relevant scales and associated stakeholders should be taken into account because stakeholder interests vary considerably across scales. Involvement beyond government administrators, policy makers and private developers has also been recommended by many (Florgård 2000; Barthel et al. 2005; Colding et al. 2006) because a significant portion of green space in cities is owned or managed by individuals or local user groups (Colding et al. 2006; Goddard et al. 2010).

Most of the reviewed studies that addressed stakeholder engagement did not include participatory methods, instead carrying out ES analysis in a top-down manner where potential consequences for stakeholders were outlined, often without linking study findings to specific planning and policy mechanisms. Turner and Daily (2008) argue that limited practical know-how of institutional design and implementation processes is a major hurdle to implementing the ES framework and propose that ES research should address every stage in the decision making process, which suggests that bottom-up research approaches are helpful not only for identifying all relevant institutional groups and structures but also to more fully integrate research into decision making, including selecting and weighting relevant ES and developing and evaluating management options. However, top-down approaches also have value, and a merging of bottom-up and top-down approaches has been suggested to more thoroughly apply the UES approach (Müller et al. 2010).

We found poor communication of research results and the exact nature of stakeholder involvement. Many papers included recommendations without any indication of the intended receivers. Additionally, none of the papers reviewed indicated how the results were fed back to the respondents or implemented in urban planning or green area management.

### Integration of UES Synergies and Trade-offs

Ecosystems deliver multiple services and can involve trade-offs that increase the provisioning of one service while reducing the provisioning of another. For example, carbon sequestration through afforestation or forest protection may enhance timber production but reduce water supplies. Such trade-offs occur if ES respond differently to changes due to temporal or spatial relationships (Seppelt et al. 2011). On the contrary, synergies between UESs entail their parallel increases or decreases (Haase et al. 2012). Often, trade-offs or synergies between ES occur unintentionally or go unnoticed (Rodríguez et al. 2006), and, when they are considered, they are frequently based on assumptions rather than findings (Carpenter et al. 2009). Integrating the assessment of multiple UESs into land management can inform decisions, making trade-offs and synergies between ES explicit and highlighting potential conflicts or win–win situations. Thus, it is very important to assess trade-offs and synergies not only to understand the system under study but also to inform policy and planning to enhance quality of life.

Of all the studies under review, 23 (10 %) considered synergies in their analysis, and 43 (20 %) mentioned trade-offs. The review analyzed trade-offs among ecosystem services and between UESs and a variety of other system components, such as land use and economic aspects. The majority of trade-offs mentioned consider the mutual relationship between UESs and land use (18). The remaining studies addressing trade-offs study trade-offs among ES (8), between ES and economic aspects (5), between ES and quality of life (2), and other trade-offs (10).

The importance of considering multiple ES as a way to address trade-offs and synergies for the purposes of planning and decision making is increasingly acknowledged, even if not often in the UES context (Buckland et al. 2005; Weber et al. 2006; Tallis and Polasky 2009; Hepcan and Ozkan 2011). Multi-criteria analysis (MCA) has been proposed as one useful methodology to analyze trade-offs and synergies. MCA is a decision support concept and methodology that enables analysis of multiple variables, which are often characterized by limited comparability (Martinez-Alier 1998). The flexibility to analyze multiples variables under the framework of MCA makes it useful for understanding and operationalizing the evaluation of social–ecological issues. MCA has been applied widely in environmental decision making (Martinez-Alier 1998). From a technical perspective, MCA methods require the scaling and ranking of variables and aggregating through weighted optimization procedures. Although there is little agreement on methods and tools for determining ranking and weighting procedures in ES MCA, there is a growing understanding that such methods are essential.

The majority of studies do not include trade-offs or synergies, although both are highly relevant for assessing different land management options and informing policy. So far, the focus has been on bio-physical science aspects, such as the relationship between land use and UESs or among multiple UESs. Particularly undervalued are trade-offs and synergies involving cultural ESs because they are subjective and difficult to quantify (Daniel et al. 2012).

## Conclusion

This review shows that studies dealing with the temporal and spatial dynamics of UESs are still rare despite their importance for urban planning. The selection of papers represents a cross-section of studies investigating ES in cities although there is more literature on non-urban ES had been published (see other ES reviews in the Electronic Supplementary Material). This review is indicative of the current state of this area. We conclude that there is a lack of both historic studies and future-oriented studies systematically analyzing the dynamics of UESs. There is also a paucity of studies based on a deep understanding of the dynamics of urban ecosystems at a more detailed level, e.g., accounting for the change of character and functionality of existing green spaces within the urban fabric. With respect to the types of ES studied, regulating, and cultural and, to a lesser degree, provisioning services were clearly emphasized. Moreover, even when several ES were studied, synergies and trade-offs between these services were not explored. Despite these limitations, current approaches to the assessment of ES dynamics include a rich array of different methodological approaches and demonstrate how these approaches can be applied to different issues relevant for urban decision making.

This review leads to a list of conclusions that are relevant for future analyses and the implementation of UES assessments. Overall, we suggest that more systematic approaches to the comprehensive assessment and evaluation of ES with a temporal dimension need to be developed both for application in retrospective studies for monitoring purposes and for future-oriented studies, in particular to support strategic planning. Future research toward integrating spatial UESs and identifying trade-offs and synergies should foster the following.

### Process Understanding, Especially the Temporal Scales

The parallel investigation of different UESs, their trade-offs and synergies, requires understanding of the processes in the system under study. A simple parallel investigation might imply statistical relationships that are merely correlations rather than causalities. Because trade-offs and synergies may vary across temporal scale, both the short-term and long-term effects of land use decisions should be evaluated and monitored to further understand the system processes and develop successful strategies (Rodríguez et al. 2006).

### A Framework to Link UES with Economic Aspects and Quality of Life

The implications of UESs for humankind manifest themselves either as synergies and trade-offs with economic aspects or with quality of life. Thus, a framework to better link UESs, economic aspects and quality of life is needed, which requires an interdisciplinary approach. There are a few frameworks available for analyzing the trade-offs of multiple ESs, but these take place at a landscape or smaller scale. Scales appropriate for urban ecosystem analysis need to be developed. There is still a great need for standardized approaches for both provisioning-side and demand-side assessments. The demand side remains largely unstudied; indicators, proxies and methods are needed. Indicators for the demand side will always include socio-economic data and are highly sensitive to demographic and population changes as well as to urban–rural mobility patterns.

### The Usage of Multi-criteria Assessment as a Tool

Many methods and models exist that can be used to integrate trade-off evaluations of ES. In particular, visualization, participatory and multi-criteria evaluation methods are promising tools for analyzing trade-offs for ecosystem services, which may include less quantifiable (especially cultural) services.

### Involving Stakeholders and Society with Different Viewpoints

An integrative view of UESs might also be fostered by involving stakeholders with different perspectives. Overall, more research is needed on how social–ecological systems generate UESs and how changes in social–ecological systems over space and time affect the provision of UESs. To make real contributions toward society and produce policy-relevant research results, knowledge about ES and values should be clearly communicated to policy makers, planners and the public. Likewise, improving UES research requires a clear and transparent presentation of participatory research methods, especially if a set of best practices for stakeholder involvement is to be developed.

### Completeness of the Regional Picture

Most of the UES studies have been undertaken in the developed West or China. Thus, there is a need to expand UES research to other parts of Asia (South Asia in particular) and South American and African countries. Because the African continent currently has the fastest urban growth rates, the utilization and conservation of UESs will be crucial for sustainably managing growth.

### Emphasizing the Concept of Ecosystem Disservices in an Urban Context

Although this review did not address disservices, and disservices were rarely addressed in the examined papers, understanding this topic could enrich our understanding of UESs and quality of life. Disservices imply a trade-off between ecosystems and quality of life. However, these trade-offs should be understood and evaluated within a local context and with a variety of stakeholders because disservices are highly subjective and variable across different environments.

### Emphasizing Spatially Explicit Approaches to UES Assessment and Valuation

Due to the well-known social and ecological heterogeneity in cities, spatially explicit UES valuation at a relatively high resolution will be critical for incorporating UES values into urban policy, planning, and management so that decisions, policies, and plans can be prioritized at the neighborhood or lot scale. Additionally, incorporating social–ecological systems theory into the application of UES valuation methods will be important for expanding our understanding of cultural services and the demand for UESs in cities, at both the local and regional scales. Given the current weak incorporation of ES into urban policy and planning in most cities, advancing spatially explicit tools in combination with multi-criteria analysis should be prioritised in UES assessment and valuation.

In closing, the concept of ES calls for an integrative assessment of the various ES that can be provided by urban nature. Furthermore, trade-offs and synergies between UES should be analyzed, and the costs and benefits of certain processes of urbanisation should be evaluated.

## Electronic supplementary material

Below is the link to the electronic supplementary material.

Supplementary material 1 (PDF 194 kb)
