# Supplementary material for: A Quantitative Review of Urban Ecosystem Service Assessments: Concepts, Models, and Implementation
Source: Ambio. 2014 Apr 17;43(4):413–33. doi: 10.1007/s13280-014-0504-0 (PMC3989520; doi:10.1007/s13280-014-0504-0)
Supplement: Supplementary file 1 — Supplementary material 1 (PDF 194 kb) [file 13280_2014_504_MOESM1_ESM.pdf]

AMBIO

Electronic Supplementary Material

A Quantitative Review of Urban Ecosystem Service Assessments: Concepts, Models and Implementation

Dagmar Haase, Neele Larondelle, Erik Andersson, Martina Artmann, Sarah Borgström, Jürgen Breuste, Erik Gomez-Baggethun, Åsa Gren, Zoé Hamstead, Rieke Hansen, Nadja Kabisch, Peleg Kremer, Johannes Langemeyer, Emily Lorance Rall, Timon McPhearson, Stephan Pauleit, Salman Qureshi, Nina Schwarz, Annette Voigt, Daniel Wurster, Jürgen Breuste, Thomas Elmqvist

List of papers reviewed

Abd-Elrahman, Amr H, Mary E Thornhill, Michael G Andreu, and Francisco Escobedo. 2010. A community-based urban forest inventory using online mapping services and consumer-grade digital images. *International Journal of Applied Earth Observation and Geoinformation* 12: 249–260. doi:10.1016/j.jag.2010.03.003.

Ahrné, K, Jan Bengtsson, and Thomas Elmqvist. 2009. Bumble Bees (Bombus spp) along a Gradient of Increasing Urbanization. *PLoS ONE* 4: 1–9. doi:10.1371/Citation.

Alberti, M. 1999. Modeling the urban ecosystem: a conceptual framework. *Environment and Planning B: Planning and Design* 26: 605–630. doi:10.1068/b260605.

Alberti, M. 2005. The Effects of Urban Patterns on Ecosystem Function. *International Regional Science Review* 28: 168–192. doi:10.1177/0160017605275160.

Alberti, Marina, John M Marzluff, Eric Shulenberger, Gordon Bradley, Craig Zumbrunnen, and Clare Ryan. 2003. Humans into Integrating and Ecology : Opportunities for Studying Urban Challenges Ecosystems. *BioScience* 53: 1169–1179.

Andersson, Erik. 2006. Urban Landscapes and Sustainable Cities. *Landscape and Urban Planning* 11.

Andersson, Erik, K Ahrné, M Pyykönen, and Thomas Elmqvist. 2009. Patterns and scale relations among urbanization measures in Stockholm, Sweden. *Landscape Ecology*: 1331–1339.

Andersson, Erik, S Barthel, and K Ahrné. 2007. Measuring scial-ecological dynamics behind the genereation of ecosystem services. *Society*: 1267–1278.

Andersson, Erik, and Örjan Bodin. 2009. Practical tool for landscape planning? An empirical investigation of network based models of habitat fragmentation. *Ecography* 32: 123–132. doi:10.1111/j.1600-0587.2008.05435.x.

Angold, P G, J P Sadler, M O Hill, a Pullin, S Rushton, K Austin, E Small, et al. 2006. Biodiversity in urban habitat patches. *The Science of the total environment* 360: 196–204. doi:10.1016/j.scitotenv.2005.08.035.

Arnfield, a. John. 2003. Two decades of urban climate research: a review of turbulence, exchanges of energy and water, and the urban heat island. *International Journal of Climatology* 23: 1–26. doi:10.1002/joc.859.

Barbosa, O, J Tratalos, P Armsworth, R Davies, R Fuller, P Johnson, and K Gaston. 2007. Who benefits from access to green space? A case study from Sheffield, UK. *Landscape and Urban Planning* 83: 187–195. doi:10.1016/j.landurbplan.2007.04.004.

Barrico, Lurdes, Anabela Marisa Azul, Maria Cristina Morais, António Pereira Coutinho, Helena Freitas, and Paula Castro. 2012. Biodiversity in urban ecosystems: Plants and macromycetes as indicators for conservation planning in the city of Coimbra (Portugal).

Landscape and Urban Planning 106. Elsevier B.V. 88–102. doi:10.1016/j.landurbplan.2012.02.011.

Barthel, Stephan, Johan Colding, Thomas Elmqvist, and Carl Folke. 2005. History and Local Management of a Biodiversity-Rich , Urban Cultural Landscape. *Ecology And Society* 10.

Barthel, Stephan, Carl Folke, and Johan Colding. 2010. Social–ecological memory in urban gardens—Retaining the capacity for management of ecosystem services. *Global Environmental Change* 20: 255–265. doi:10.1016/j.gloenvcha.2010.01.001.

Baumgardner, Darrel, Sebastian Varela, Francisco J Escobedo, Alicia Chacalo, and Carlos Ochoa. 2012. The role of a peri-urban forest on air quality improvement in the Mexico City megalopolis. *Environmental pollution (Barking, Essex : 1987)* 163. Elsevier Ltd: 174–83. doi:10.1016/j.envpol.2011.12.016.

Van den Berg, Agnes E, T. Harting, and H. Staats. 2007. Preference for Nature in Urbanized Societies : Stress , Restoration , and the Pursuit of Sustainability. *Journal of Social Issues* 63: 79–96.

Van den Berg, Agnes E, Jolanda Maas, Robert a Verheij, and Peter P Groenewegen. 2010. Green space as a buffer between stressful life events and health. *Social science & medicine (1982)* 70. Elsevier Ltd: 1203–10. doi:10.1016/j.socscimed.2010.01.002.

Blair, R B. 1999. BIRDS AND BUTTERFLIES ALONG AN URBAN GRADIENT: SURROGATE TAXA FOR ASSESSING BIODIVERSITY? *Ecological Applications* 9: 164–170.

Blair, R B. 2004. The Effects of Urban Sprawl on Birds at Multiple Levels of Biological Organization. *Ecology and Society* 9.

Bolund, P, and S Hunhammar. 1999. Ecosystem Services in urban areas. *Ecological Economics* 29: 293–301.

Borgström, S., S.a.O. Cousins, and R. Lindborg. 2012. Outside the boundary – Land use changes in the surroundings of urban nature reserves. *Applied Geography* 32. Elsevier Ltd: 350–359. doi:10.1016/j.apgeog.2011.06.012.

Bowler, Diana E., Lisette Buyung-Ali, Teri M. Knight, and Andrew S. Pullin. 2010. Urban greening to cool towns and cities: A systematic review of the empirical evidence. *Landscape and Urban Planning* 97. Elsevier B.V. 147–155. doi:10.1016/j.landurbplan.2010.05.006.

Boyer, Tracy, and Stephen Polasky. 2004. valuing urban wetlands: a review of non-market valuations studies. *Society of wetland scientist* 24: 744–755.

Brack, C L. 2002. Pollution mitigation and carbon sequestration by an urban forest. *Environmental Pollution* 116: 195–200.

Breuste, J H. 2004. Decision making, planning and design for the conservation of indigenous vegetation within urban development. *Landscape and urban Planning* 68: 439–452.

Burkhard, B, F Kroll, F Müller, and W Windhorst. 2009. Landscapes´Capacities to Provide Ecosystem Services - a Concept for Land-Cover Based Assessments. *Landscape Online*: 1–22.

Burkhard, Benjamin, Franziska Kroll, Stoyan Nedkov, and Felix Müller. 2011. Mapping ecosystem service supply, demand and budgets. *Ecological Indicators* in Press. Elsevier Ltd. doi:10.1016/j.ecolind.2011.06.019.

Chee, Y E. 2004. An ecological perspective on the valuation of ecosystem services. *Biological Conservation* 120: 549–565.

Chiesura, A. 2004. The role of urban parks for the sustainable city. *Landscape and urban Planning* 68: 129–138.

Cho, S, N Poudyal, and R Roberts. 2008. Spatial analysis of the amenity value of green open space. *Ecological Economics* 66: 403–416. doi:10.1016/j.ecolecon.2007.10.012.

Choumert, Johanna, and Laure Cormier. 2010. The provision of urban parks: an empirical test of spatial spillovers in an urban area using geographic information systems. *The Annals of Regional Science* 47: 437–450. doi:10.1007/s00168-010-0366-8.

Clergeau, P, S Croci, J Jokimäki, M.-L. Kaisanlahti-Jokimäki, and M Dinetti. 2006. Avifauna homogenisation by urbanisation: Analysis at different European latitudes. *Biological Conservation* 127: 336–344.

Clergeau, P, J.-P. Savard, G Mennechez, and G Falardeau. 1998. Bird abundance and diversity along an urban-rural gradient: A comparative study between two cities on different continents. *The Condor* 100: 413–425.

Colding, J. 2007. "Ecological land-use complementation" for building resilience in urban ecosystems. *Landscape and Urban Planning* 81: 46–55. doi:10.1016/j.landurbplan.2006.10.016.

Coley, R. L., W. C. Sullivan, and F. E. Kuo. 1997. Where Does Community Grow?: The Social Context Created by Nature in Urban Public Housing. *Environment and Behavior* 29: 468–494. doi:10.1177/001391659702900402.

Cook, E. 2002. Landscape structure indices for assessing urban ecological networks. *Landscape and Urban Planning* 58: 269–280. doi:10.1016/S0169-2046(01)00226-2.

Cornelis, J, and M Hermy. 2004. Biodiversity relationships in urban and suburban parks in Flanders. *Landscape and urban Planning* 69: 385–401.

Costanza, Robert, William J Mitsch, and J W Day Jun. 2012. A New Vision for New Orleans and the Mississippi Delta : Applying Ecological Economics and Ecological Engineering. *Ecological Engineering* 4: 465–472.

Dale, V H, A W King, L K Mann, R A Washington-Allen, and R A McCord. 1998. Assessing Land-Use Impacts on Natural Resources. *Environmental Management* 22: 203–211.

Dixon, Timothy H, Falk Amelung, Alessandro Ferretti, Fabrizio Novali, Fabio Rocca, Roy Dokka, Giovanni Sella, Sang-Wan Kim, Shimon Wdowinski, and Dean Whitman. 2006. Subsidence and flooding in New Orleans. *Nature* 441: 587–8. doi:10.1038/441587a.

Dobbs, Cynnamon, Francisco J Escobedo, and Wayne C Zipperer. 2011. A framework for developing urban forest ecosystem services and goods indicators. *Landscape and Urban Planning* 99. Elsevier B.V. 196–206. doi:10.1016/j.landurbplan.2010.11.004.

Dow, Kirstin. 2002. Social dimensions of gradients in urban ecosystems. *Urban Ecosystems* 4: 255–275.

Dwyer, J F, E G McPherson, H W Schroeder, and R A Rowntree. 1992. Assessing the benefits and costs of the urban forest. *Journal of Arboriculture* 18: 227–234.

Dwyer, Mark C., and Robert W Miller. 1999. Using GIS to assess urban tree canopy benefits and surrounding greenspace distributions. *Journal of Arboriculture* 25: 102–107.

Dye, Christopher. 2008. Health and urban living. *Science (New York, N.Y.)* 319: 766–9. doi:10.1126/science.1150198.

Elliott, a, and S Trowsdale. 2007. A review of models for low impact urban stormwater drainage. *Environmental Modelling & Software* 22: 394–405. doi:10.1016/j.envsoft.2005.12.005.

Elmqvist, T, C Alfsen, and J Colding. 2008. Urban systems. In *Encyclopedia of Ecology*, ed. S. E. Jorgensen and B. D. Fath, 5:3665–3672. Elsevier Oxford.

Elnaboulsi, Jihad C. 2011. An Efficient Pollution Control Instrument: The Case of Urban Wastewater Pollution. *Environmental Modeling & Assessment* 16: 343–358. doi:10.1007/s10666-011-9261-7.

Eppink, F V, J.C.J.M. van den Bergh, and P Rietveld. 2004. Modelling biodiversity and land use: urban growth, agriculture and nature in a wetland area. *Ecological Economics* 51: 201–216.

Ernstson, Henrik, Sander E Van Der Leeuw, Charles L Redman, J Douglas, George Davis, Christine Alfsen, and Thomas Elmqvist. 2009. Urban transitions : on urban resilience and human-dominated ecosystems Authors. *Ambio* 1: 1–22.

Ernstson, Henrik, S Sörlin, and Thomas Elmqvist. 2008. Social Movements and Ecosystem Services—the Role of Social Network Structure in Protecting and Managing Urban Green Areas in Stockholm. *Ecology And Society* 13: 39.

Escobedo, Francisco J, Timm Kroeger, and John E Wagner. 2011. Urban forests and pollution mitigation: Analyzing ecosystem services and disservices. *Environmental Pollution* 159. Elsevier Ltd: 2078–2087. doi:10.1016/j.envpol.2011.01.010.

Escobedo, Francisco J, and David J Nowak. 2009. Spatial heterogeneity and air pollution removal by an urban forest. *Landscape and Urban Planning* 90: 102–110.

Escobedo, Francisco J, John E Wagner, David J Nowak, Carmen Luz De la Maza, Manuel Rodriguez, and Daniel E Crane. 2008. Analyzing the cost effectiveness of Santiago, Chile's policy of using urban forests to improve air quality. *Journal of Environmental Management* 86: 148–157.

Fanger, P O. 1973. Assessment of man's thermal comfort in practice. *British journal of industrial medicine* 30: 313–24.

Findlay, Sophia, Mark Taylor, Peter Davies, and Amylia Fletcher. 2011. Development and application of a rapid assessment tool for urban stream networks. *Water and Environment Journal* 25: 2–12. doi:10.1111/j.1747-6593.2009.00178.x.

Fitzhugh, Thomas W, and Brian D Richter. 2004. Quenching Urban Thirst : Growing Cities and Their Impacts on Freshwater Ecosystems. *BioScience* 54.

Florgård, Clas. 2000. Long-term changes in indigenous vegetation preserved in urban areas. *Landscape and urban Planning* 52: 101–116.

Foley, J A, R DeFries, C Asner, C Barford, G Bonan, S R Carpenter, F S Chapin III, et al. 2005. Global Consequences of Land Use. *Science* 309: 570–574.

Folke, Carl, Asa Jansson, Jonas Larsson, and Robert Costanza. 1997. Ecosystem Appropiation by Cities. *AMBIO* 26: 167–172.

Fuller, R A, and K J Gaston. 2010. The scaling of green space coverage in European cities. *Biology Letters*.

Fuller, Richard a, Katherine N Irvine, Patrick Devine-Wright, Philip H Warren, and Kevin J Gaston. 2007. Psychological benefits of greenspace increase with biodiversity. *Biology letters* 3: 390–4. doi:10.1098/rsbl.2007.0149.

Gill, S E, J F Handley, A R Ennos, and S Pauleit. 2007. Adapting Cities for Climate Change: The Role of the Green Infrastructure. *Built Environment* 33: 115–133.

Gill, S E, J F Handley, A R Ennos, S Pauleit, N Theuray, and S J Lindley. 2008. Characterising the urban environment of UK cities and towns: A template for landscape planning. *Landscape and urban Planning* 87: 210–222.

Givoni, B. 1991. Impact of planted areas on urban environmental quality: A review. *Atmospheric Environment. Part B. Urban Atmosphere* 25: 289–299. doi:10.1016/0957-1272(91)90001-U.

Goddard, Mark a, Andrew J Dougill, and Tim G Benton. 2010. Scaling up from gardens: biodiversity conservation in urban environments. *Trends in ecology & evolution* 25: 90–8. doi:10.1016/j.tree.2009.07.016.

Godefroid, S, and N Koedam. 2003. Distribution pattern of the flora in a peri-urban forest: an effect of the city - forest ecotone. *Landscape and urban Planning* 65: 169–185.

Grimmond, C Susan B, and T R Oke. 1999. Heat Storage in Urban Areas : Local-Scale Observations and Evaluation of a Simple Model. *Journal of Applied Meteorology*: 922–940.

Grove, J M, Austin R Troy, J.P.M. O´Neil-Dunne, W.R. Burch jun., M. L. Cadenasso, and S. T. Pickett. 2006. Characterization of Housholds and its Implications for the Vegetation of Urban Ecosystems. *Ecosystems* 9: 578–597.

Haase, D. 1997. Urban Ecology in the new federal countries of Germany. Contamination of soil and urban atmosphere with heavy metals in Leipzig. *Arch.für Nat.- Lands.* 37: 45–58.

Haase, D. 2008. Modelling the effects of long-term urban land use change on the water balance. *AGD Landscape & Environment* 2: 144–160.

Haase, Dagmar. 2009. Effects of urbanisation on the water balance – A long-term trajectory. *Environmental Impact Assessment Review* 29: 211–219. doi:10.1016/j.eiar.2009.01.002.

Herbst, H, and V Herbst. 2006. The development of an evaluation method using a geographic information system to determine the importance of wasteland sites as urban wildlife areas. *Landscape and urban Planning* 77: 178–195.

Heynen, N C. 2003. The Scalar Production of Injustice within the Urban Forest. *Antipode*: 980–998.

Holt, A R, and C Hattam. 2009. Capitalizing on nature: how to implemet an ecosytsem approach. *Biology Letters* 5: 580–582.

Hong, Bongghi, Karin E Limburg, Jon D Erickson, John M Gowdy, Audra A Nowosielski, John M Polimeni, and Karen M Stainbrook. 2009. Connecting the ecological-economic dots in human-dominated watersheds: Models to link socio-economic activities on the landscape to stream ecosystem health. *Landscape and Urban Planning* 91: 78–87. doi:10.1016/j.landurbplan.2008.11.012.

Hope, D, C Gries, W Zhu, W F Fagan, C L Redman, N B Grimm, A L Nelson, C Martin, and A Kinzig. 2003. Socioeconomics drive urban plant diversity. *PNAS* 100: 8788–8792.

Hougner, Cajsa, Johan Colding, and Tore Söderqvist. 2005. Economic valuation of a seed dispersal service in the Stockholm National Urban Park , Sweden. *Ecological Economics* 9: 364–374.

Hunter, Mary Carol R., and Daniel G. Brown. 2012. Spatial contagion: Gardening along the street in residential neighborhoods. *Landscape and Urban Planning* 105. Elsevier B.V. 407–416. doi:10.1016/j.landurbplan.2012.01.013.

Iamtrakul, Pawinee. 2005. Public park valuation using travel cost method 5: 1249–1264.

Imhoff, M L, L Bounoua, R DeFries, W T Lawrence, D Stutzer, C J Tucker, and T Ricketts. 2004. The consequences of urban land transformation on net primary productivity in the United States. *Remote Sensing of Environment* 89: 434–443.

Iverson, Louis R, and Elizabeth A Cook. 2001. Urban forest cover of the Chicago region and its relation to household density and income. *Rivers* 4: 105–124.

James, P., K. Tzoulas, M.D. Adams, A. Barber, J. Box, J. Breuste, T. Elmqvist, M. Frith, C. Gordon, and K.L. Greening. 2009. Towards an integrated understanding of green space in the European built environment. *Urban Forestry & Urban Greening* 8: 65–75. doi:10.1016/j.ufug.2009.02.001.

Jansson, A, and P Nohrstedt. 2001. Carbon sinks and human freshwater dependence in Stockholm County. *Ecological Economics* 39: 361–370.

Jansson, Asa, and Johan Colding. 2007. Tradeoffs between environmental goals and urban development: the case of nitrogen load from the Stockholm County to the Baltic Sea. *Ambio* 36: 650–6.

Jansson, Asa, and Steven Polasky. 2010. Quantifying Biodiversity for Building Resilience for Food Security in Urban Landscapes : Getting Down to Business. *Ecology And Society* 15.

Jenerette, G Darrel, Wendy A Marussich, and Joshua P Newell. 2005. Linking ecological footprints with ecosystem valuation in the provisioning of urban freshwater. *Urban Water* 59: 38–47.

Jim, C Y, and W Y Chen. 2009. Ecosystem services and valuation of urban forests in China. *Cities* 26: 187–194.

Jim, C Y, and Wendy Y Chen. 2008. Assessing the ecosystem service of air pollutant removal by urban trees in Guangzhou (China). *Journal of Environmental Management* 88: 665–676.

Jim, C.Y., and Wendy Y. Chen. 2006. Impacts of urban environmental elements on residential housing prices in Guangzhou (China). *Landscape and Urban Planning* 78: 422–434. doi:10.1016/j.landurbplan.2005.12.003.

Jo, H., and G. McPherson. 1995. Carbon storage and flux in urban residential greenspace. *Journal of Environmental Management* 45: 109–133.

Jujnovsky, Julieta, Teresa Margarita González-Martínez, Enrique Arturo Cantoral-Uriza, and Lucia Almeida-Leñero. 2012. Assessment of water supply as an ecosystem service in a rural-urban watershed in southwestern Mexico City. *Environmental management* 49: 690–702. doi:10.1007/s00267-011-9804-3.

Kliskey, A D. 2000. Recreation terrain suitability mapping: a spatially explicit methodology for determining recreation potential for resource use assessment. *Landscape and Urban Planning* 52: 33–43. doi:10.1016/S0169-2046(00)00111-0.

Knapp, Sonja, Ingolf Kühn, Jan P. Bakker, Michael Kleyer, Stefan Klotz, Wim a. Ozinga, Peter Poschlod, Ken Thompson, Wilfried Thuiller, and Christine Römermann. 2009. How species traits and affinity to urban land use control large-scale species frequency. *Diversity and Distributions* 15: 533–546. doi:10.1111/j.1472-4642.2009.00561.x.

Knapp, Sonja, Ingolf Kühn, Oliver Schweiger, and Stefan Klotz. 2008. Challenging urban species diversity: contrasting phylogenetic patterns across plant functional groups in Germany. *Ecology letters* 11: 1054–64. doi:10.1111/j.1461-0248.2008.01217.x.

Kong, Fanhua, Haiwei Yin, and Nobukazu Nakagoshi. 2007. Using GIS and landscape metrics in the hedonic price modeling of the amenity value of urban green space: A case study in Jinan City, China. *Landscape and Urban Planning* 79: 240–252. doi:10.1016/j.landurbplan.2006.02.013.

Konijnendijk, Cecil C. 2003. A decade of urban forestry in Europe. *Forest Policy and Economics* 5: 173–186. doi:10.1016/S1389-9341(03)00023-6.

Kremer, Peleg, and Tracy L. DeLiberty. 2011. Local food practices and growing potential: Mapping the case of Philadelphia. *Applied Geography* 31: 1252–1261. doi:10.1016/j.apgeog.2011.01.007.

Kreuter, Urs P, Heather G Harris, Marty D Matlock, and Ronald E Lacey. 2001. Change in ecosystem service values in the San Antonio area, Texas. *Ecological Economics* 39: 333–346. doi:10.1016/S0921-8009(01)00250-6.

Kroll, Franziska, Felix Müller, Dagmar Haase, and Nicola Fohrer. 2011. Rural–urban gradient analysis of ecosystem services supply and demand dynamics. *Land Use Policy*. Elsevier Ltd. doi:10.1016/j.landusepol.2011.07.008.

Kühn, I, and S Klotz. 2006. Urbanization and homogenization - Comparing the floras of urban and rural areas in Germany. *Biological Conservation* 127: 292–300.

Lafortezza, Raffaele, Giuseppe Carrus, Giovanni Sanesi, and Clive Davies. 2009. Benefits and well-being perceived by people visiting green spaces in periods of heat stress. *Urban Forestry & Urban Greening* 8: 97–108. doi:10.1016/j.ufug.2009.02.003.

Lakes, Tobia, and Hyun-Ok Kim. 2012. The urban environmental indicator "Biotope Area Ratio"—An enhanced approach to assess and manage the urban ecosystem services using high resolution remote-sensing. *Ecological Indicators* 13. Elsevier Ltd: 93–103. doi:10.1016/j.ecolind.2011.05.016.

Lal, R. 2004. Review Soil carbon sequestration to mitigate climate change. *Geoderma* 123: 1–22.

Li, F, R Wang, J Paulussen, and X Liu. 2005. Comprehensive concept planning of urban greening based on ecological principles: a case study in Beijing, China. *Landscape and urban Planning* 72: 325–336.

Liu, Changfu, and Xiaoma Li. 2012. Carbon storage and sequestration by urban forests in Shenyang, China. *Urban Forestry & Urban Greening* 11. Elsevier GmbH. 121–128. doi:10.1016/j.ufug.2011.03.002.

Liu, G, Z Yang, B Chen, Y Zhang, L Zhang, Y Zhao, and M Jiang. 2009. Emergy-based urban ecosystem health assessment: A case study of Baotou, China. *Communications in Nonlinear Science and Numerical Simulation* 14: 972–981. doi:10.1016/j.cnsns.2007.09.017.

Liu, Yong, Jinchang Li, and Hong Zhang. 2012. An ecosystem service valuation of land use change in Taiyuan City, China. *Ecological Modelling* 225. Elsevier B.V. 127–132. doi:10.1016/j.ecolmodel.2011.11.017.

Lockaby, B G, D Zhang, H Tian, and S Pan. 2005. Interdisciplinary research at the Urban – Rural interface : The WestGa project. *Urban Ecosystem* 8: 7–21.

Locke, Dexter H, J Morgan Grove, Jacqueline W T Lu, Austin Troy, Jarlath P M O Neil-dunne, and Brian D Beck. 2010. Prioritizing Preferable Locations for Increasing Urban Tree Canopy in New York City. *Cities and the Environment* 3: 1–18.

Longcore, Travis, Christina Li, and John P. Wilson. 2004. Applicability of CityGreen urban ecosystem analysis software to a densely built urban neighborhood. *Urban Geography* 25: 173–186.

Lorance Rall, E D, and D Haase. 2010. Creative intervention in a dynamic city:A sustainability assessment of an interim use strategy for brownfields inLeipzig,Germany. *Landscape and urban Planning*.

Luck, Matthew a., G. Darrel Jenerette, Jianguo Wu, and Nancy B. Grimm. 2001. The Urban Funnel Model and the Spatially Heterogeneous Ecological Footprint. *Ecosystems* 4: 782–796. doi:10.1007/s10021-001-0046-8.

Lundberg, Jakob, Erik Andersson, Grainne Cleary, and Thomas Elmqvist. 2008. Linkages beyond borders: targeting spatial processes in fragmented urban landscapes. *Landscape Ecology* 23: 717–726. doi:10.1007/s10980-008-9232-9.

Lundy, L., and R. Wade. 2011. Integrating sciences to sustain urban ecosystem services. *Progress in Physical Geography* 35: 653–669. doi:10.1177/0309133311422464.

Maas, Jolanda, Robert a Verheij, Peter P Groenewegen, Sjerp de Vries, and Peter Spreeuwenberg. 2006. Green space, urbanity, and health: how strong is the relation? *Journal of epidemiology and community health* 60: 587–92. doi:10.1136/jech.2005.043125.

Mace, Georgina M., Ken Norris, and Alastair H. Fitter. 2011. Biodiversity and ecosystem services: a multilayered relationship. *Trends in Ecology & Evolution* 27: 19–26. doi:10.1016/j.tree.2011.08.006.

Makinen, K, and L Tyrvainen. 2008. Teenage experiences of public green spaces in suburban Helsinki. *Urban Forestry & Urban Greening* 7: 277–289. doi:10.1016/j.ufug.2008.07.003.

Mäler, K.-G., S Aniyar, and A Jansson. 2008. Accounting for ecosystem services as a way to understand the requirements for sustainable development. *PNAS* 28: 9501–9506.

Matsuoka, R H, and R Kaplan. 2008. People needs in the urban landscape: Analysis of Landscape And Urban Planning contributions. *Landscape and urban Planning* 84: 7–19.

Mayer, H., A. Matzarakis, and M. G. Iziomon. 2003. Spatio-temporal variability of moisture conditions within the Urban Canopy Layer. *Theoretical and Applied Climatology* 76: 165–179. doi:10.1007/s00704-003-0010-y.

McDonald, R I. 2008. Global urbanization: can ecologists identify a sustainable way forward? *Frontiers in Ecology and the Environment* 6.

McDonald, R I. 2009. Ecosystem service demand and supply along the urban-to-rural gradient. *Journal of Conservation Planning* 5: 1–14.

McKinney, M L. 2002. Urbanization, Biodiversity, and Conservation. *BioScience* 52: 883–890.

McLain, Rebecca, Melissa Poe, Patrick T. Hurley, Joyce Lecompte-Mastenbrook, and Marla R. Emery. 2012. Producing edible landscapes in Seattle's urban forest. *Urban Forestry & Urban Greening* 11. Elsevier GmbH. 187–194. doi:10.1016/j.ufug.2011.12.002.

McPherson, E G. 1992. Accounting for benefits and costs of urban greenspace. *Landscape and urban Planning* 22: 41–51.

McPherson, E Gregory. 1998. Atmospheric carbon dioxide reduction by Sacramento's urban forest. *Journal Of Arboriculture* 24: 215–223.

McPherson, E Gregory, David J Nowak, and Rowan A Rowntree. 1994. *Chicago's Urban Forest Ecosystem: Results of the Chicago Urban Forest Climate Project. Urban Ecosystems*.

Mcpherson, E Gregory, and Rowan A Rowntree. 1993. energy conservation potential of urban tree planting. *Journal of Arboriculture* 19: 321–331.

Mcpherson, E Gregory, and James R. Simpson. 2003. Potential energy savings in buildings by an urban tree planting programme. *Urban Forestry & Urban Greening2* 2: 73–86.

McPherson, E. G., D. Nowak, G. Heisler, S. Grimmond, C. Souch, R. Grant, and R. Rowntree. 1997. Quantifying urban forest structure, function, and value: the Chicago urban forest climate project. *Urban Ecosystems* 1: 49–61.

McPherson, Gregory E, James R Simpson, Paula J. Peper, and Qingfu Xiao. 1999. Benefit-cost analysis of modesto´s municipal forest. *Journal of Arboriculture* 25: 235–248.

Melles, S J. 2005. Urban Bird Diversity as an Indicator of Human Social Diversity and Economic Inequality in Vancouver, British Columbia. *Urban Habitats* 3: 25–48.

Morani, Arianna, David J Nowak, Satoshi Hirabayashi, and Carlo Calfapietra. 2011. How to select the best tree planting locations to enhance air pollution removal in the MillionTreesNYC initiative. *Environmental Pollution* 159: 1040–1047.

More, Thomas a., Thomas Stevens, and P.Geoffrey Allen. 1988. Valuation of urban parks. *Landscape and Urban Planning* 15: 139–152. doi:10.1016/0169-2046(88)90022-9.

Mörtberg, U, and H.-G. Wallentinus. 2000. Red-listed forest bird species in an urban environment - assessment of green space corridors. *Landscape and urban Planning* 50: 215–226.

Müller, Felix, Rudolf de Groot, and Louise Willemen. 2010. Ecosystem Services at the Landscape Scale: the Need for Integrative Approaches. *Landscape Online* 23: 1–11. doi:10.3097/LO.201023.

Muratet, A, N Machon, F Jiguet, J Moret, and E Porcher. 2007. The Role of Urban Structures in the Distribution of Wasteland Flora in the Greater Paris Area, France. *Ecosystems* 10: 661–671.

Myeong, S, D Nowak, and M J Duggin. 2006. A temporal analysis of urban forest carbon storage using remote sensing. *Remote Sensing of Environment* 101: 277–282.

Naizheng, Xu, Liu Hongying, Wei Feng, and Zhu Yiping. 2011. Urban expanding pattern and soil organic, inorganic carbon distribution in Shanghai, China. *Environmental Earth Sciences* 66: 1233–1238. doi:10.1007/s12665-011-1334-z.

Natuhara, Y, and C Imai. 1996. Spatial structure of avifauna along urban-rural gradients. *Ecological Research* 11: 1–9.

Nelson, E, S Polasky, D J Lewis, A J Plantinga, E Lonsdorf, D White, D Dael, and J J Lawler. 2008. Efficiency of incentives to jointly increase carbon sequestration and species conservation on a landscape. *PNAS* 28: 9471–9476.

Niemelä, J, S.-R. Saarela, T Söderman, L Kopperoinen, V Yli-Pelkonen, S Väre, and D J Kotze. 2010. Using the ecosystem services approach for better planning and conservation of urban green spaces: a Finland case study. *Biodiversity Conservation* 19: 3225–3243.

Nowak, D, and D E Crane. 1998. The Urban Forest Effects (UFORE) Model: Quantifying Urban Forest Structure and Functions. *Integrated Tools Proceedings*: 714–720.

Nowak, D, and D E Crane. 2002. Carbon storage and sequestration by urban trees in the USA. *Environmental Pollution* 116: 381–389.

Nowak, D, J C Stevens, S M Sisinni, and C J Luley. 2002. Effects of urban tree management and species selection on atmospheric carbon dioxide. *Journal of Arboriculture* 28: 122–133.

Nowak, David J, Daniel E Crane, and Jack C Stevens. 2006. Air pollution removal by urban trees and shrubs in the United States. *Urban Forestry & Urban Greening* 4: 115–123.

Nuissl, H, D Haase, M Lanzendorf, and H Wittmer. 2009. Environmental impact assessment of urban land use transitions. A context-sensitive approach. *Land Use Policy* 26: 414–424.

Oberndorfer, Erica, Jeremy Lundholm, Brad Bass, Reid R. Coffman, Hitesh Doshi, Nigel Dunnett, Stuart Gaffin, Manfred Köhler, Karen K. Y. Liu, and Bradley Rowe. 2007. Green Roofs as Urban Ecosystems: Ecological Structures, Functions, and Services. *BioScience* 57: 823. doi:10.1641/B571005.

Palmer, M, E Bernhardt, E Chornesky, S Collins, A Dobson, C Duke, and B Gold. 2004. Ecology for a Crowded Planet: Policy Forum. *Science* 304: 1251–1252.

Paoletti, Elena. 2009. Ozone and urban forests in Italy. *Environmental Pollution* 157: 1506–1512.

Pataki, D. E., R. J. Alig, a. S. Fung, N. E. Golubiewski, C. a. Kennedy, E. G. Mcpherson, D. J. Nowak, R. V. Pouyat, and P. Romero Lankao. 2006. Urban ecosystems and the North American carbon cycle. *Global Change Biology* 12: 2092–2102. doi:10.1111/j.1365-2486.2006.01242.x.

Pataki, Diane E, Margaret M Carreiro, Jennifer Cherrier, Nancy E Grulke, Viniece Jennings, Stephanie Pincetl, Richard V Pouyat, Thomas H Whitlow, and Wayne C Zipperer. 2011. Coupling biogeochemical cycles in urban environments: ecosystem services, green solutions, and misconceptions. *Frontiers in Ecology and the Environment* 9: 27–36. doi:10.1890/090220.

Pellissier, Vincent, Audrey Muratet, Fabien Verfaillie, and Nathalie Machon. 2012. Pollination success of Lotus corniculatus (L.) in an urban context. *Acta Oecologica* 39. Elsevier Masson SAS: 94–100. doi:10.1016/j.actao.2012.01.008.

Pickett, Steward T A, Mary L Cadenasso, J Morgan Grove, Peter M Groffman, Lawrence E Band, Christopher G Boone, William R Burch, et al. 2008. Beyond Urban Legends: An Emerging Framework of Urban Ecology, as Illustrated by the Baltimore Ecosystem Study. *American Institute of Biological Sciences BioScience* 58: 139–150.

Poudyal, Neelam C., Donald G. Hodges, and Christopher D. Merrett. 2009. A hedonic analysis of the demand for and benefits of urban recreation parks. *Land Use Policy* 26: 975–983. doi:10.1016/j.landusepol.2008.11.008.

Pouyat, R, P Groffman, I Yesilonis, and L Hernandez. 2002. Soil carbon pools and fluxes in urban ecosystems. *Environmental Pollution* 116: 107–118.

Pouyat, Richard V, Ian D Yesilonis, and David J Nowak. 2006. Carbon Storage by Urban Soils in the United States. *Journal of Environmental Quality* 35: 1566–1575. doi:10.2134/jeq2005.0215.

Rosenzweig, Cynthia, William Solecki, Lily Parshall, Stuart Gaffin, Barry Lynn, Richard Goldberg, Jennifer Cox, and Sara Hodges. 2006. Mitigating New York City's heat island with urban forestry, living roofs, and light surfaces. In *Proceedings of the Sixth Symposium on the Urban Environment*. Atlanta, GA.

Rowntree, R A, and D Nowak. 1991. QUANTIFYING THE ROLE OF URBAN FORESTS IN REMOVING ATMOSPHERIC CARBON DIOXIDE. *Journal of Arboriculture* 17: 269–275.

Sander, Heather, Stephen Polasky, and Robert G. Haight. 2010. The value of urban tree cover: A hedonic property price model in Ramsey and Dakota Counties, Minnesota, USA. *Ecological Economics* 69. Elsevier B.V. 1646–1656. doi:10.1016/j.ecolecon.2010.03.011.

Schetke, S, and D Haase. 2008. Multi-criteria assessment of socio-environmental aspects in shrinking cities. Experiences from eastern Germany. *Environmental Impact Assessment Review* 28: 483–503.

Schetke, Sophie. 2010. Socio-environmental impacts of settlement growth under conditions of fostered infill development: a methodological framework for a multicriteria assessment. Bonn: Geodäsie und Geoinformation.

Schetke, Sophie, Dagmar Haase, and Theo Kötter. 2012. Towards sustainable settlement growth: A new multi-criteria assessment for implementing environmental targets into strategic urban planning. *Environmental Impact Assessment Review* 32. Elsevier Inc. 195–210. doi:10.1016/j.eiar.2011.08.008.

Schwarz, N, A Bauer, and D Haase. 2011. Assessing climate impacts of planning policies: An estimation for the urban region of Leipzig (Germany). *Environmental Impact Assessment Review* 31: 97–111.

Schwarz, Nina, Sven Lautenbach, and Ralf Seppelt. 2011. Exploring indicators for quantifying surface urban heat islands of European cities with MODIS land surface temperatures. *Remote Sensing of Environment* 115: 3175–3186. doi:10.1016/j.rse.2011.07.003.

Shashua-bar, L, and M E Hoffman. 2000. Vegetation as a climatic component in the design of an urban street An empirical model for predicting the cooling effect of urban green areas with trees. *Energy and Buildings* 31: 221–235.

Sherrouse, Benson C., Jessica M. Clement, and Darius J. Semmens. 2011. A GIS application for assessing, mapping, and quantifying the social values of ecosystem services. *Applied Geography* 31. Elsevier Ltd: 748–760. doi:10.1016/j.apgeog.2010.08.002.

Silaydin Aydin, Mediha Burcu, and Duygu Çukur. 2011. Maintaining the carbon–oxygen balance in residential areas: A method proposal for land use planning. *Urban Forestry & Urban Greening*. doi:10.1016/j.ufug.2011.09.008.

Simpson, James R. 2002. Improved estimates of tree-shade effects on residential energy use. *Energy* 34.

Snep, R P H, P F M Opdam, J M Baveco, M F WallisDeVries, W Timmermans, R G M Kwak, and V Kuypers. 2006. How peri-urban areas can strengthen animal populations within cities: A modeling approach. *Biological Conservation* 127: 345–355.

Soares, A L, F C Rego, E Gregory McPherson, J R Simpson, P J Peper, and Q Xiao. 2011. Benefits and costs of street trees in Lisbon, Portugal. *Urban Forestry & Urban Greening* 10. Elsevier GmbH. 69–78. doi:10.1016/j.ufug.2010.12.001.

Stratford, Jefrey A., and W Douglas Robinson. 2005. Distribution of neotropical migratory bird species across an urbanizing landscape. *Urban Ecosystems* 8: 59–77.

Strauss, B, and R Bidermann. 2006. Urban brownfields as temporary habitats: driving forces for the diversity of phytophagous insects. *Ecography* 29: 928–940.

Strohbach, M W, and D Haase. 2010. Estimating the carbon stock of a city: a study from Leipzig, Germany. *Landscape and urban Planning*.

Strohbach, M W, D Haase, and N Kabisch. 2009. Research - Birds and the City: Urban Biodiversity, Land Use, and Socioeconomics. *Ecology and Society* 14.

Strohbach, Michael W, Eric Arnold, and Dagmar Haase. 2012. The carbon footprint of urban green space - A life cycle approach. *Landscape and Urban Planning* 104: 220–229.

Strohbach, Michael W, and Dagmar Haase. 2012. Above-ground carbon storage by urban trees in Leipzig, Germany: Analysis of patterns in a European city. *Landscape and Urban Planning* 104. Elsevier B.V. 95–104. doi:10.1016/j.landurbplan.2011.10.001.

Su, M, Z Yang, and B Chen. 2010. Ecosystem health pattern analysis of urban clusters - A comparative study of three typical Chinese urban clusters. *science of the total environment*.

Su, M.R., Z.F. Yang, and B. Chen. 2009. Set pair analysis for urban ecosystem health assessment. *Communications in Nonlinear Science and Numerical Simulation* 14. Elsevier B.V. 1773–1780. doi:10.1016/j.cnsns.2007.07.019.

Su, M.R., Z.F. Yang, B. Chen, and S. Ulgiati. 2009. Urban ecosystem health assessment based on emergy and set pair analysis—A comparative study of typical Chinese cities. *Ecological Modelling* 220: 2341–2348. doi:10.1016/j.ecolmodel.2009.06.010.

Su, Meirong, and Brian D. Fath. 2012. Spatial distribution of urban ecosystem health in Guangzhou, China. *Ecological Indicators* 15. Elsevier Ltd: 122–130. doi:10.1016/j.ecolind.2011.09.040.

Tallis, Matthew, Gail Taylor, Danielle Sinnett, and Peter Freer-Smith. 2011. Estimating the removal of atmospheric particulate pollution by the urban tree canopy of London, under current and future environments. *Landscape and Urban Planning* 103: 129–138.

Taylor, Publisher, Wendy Y Chen, and C Y Jim. 2012. Contingent valuation of ecotourism development in country parks in the urban shadow. *International Journal of Sustainable Development & World Ecology* 19: 44–53.

Tiwary, A, D Sinnett, C Peachey, Z Chalabi, S Vardoulakis, and T Fletcher. 2009. An integrated tool to assess the role of new planting in PM10 capture and the human health benefits: A case study in London. *Environmental Pollution* 157: 2645–2653.

Tong, Chunfu, Rusty a. Feagin, Jianjian Lu, Xiufeng Zhang, Xiaojun Zhu, Wei Wang, and Wenshan He. 2007. Ecosystem service values and restoration in the urban Sanyang wetland of Wenzhou, China. *Ecological Engineering* 29: 249–258. doi:10.1016/j.ecoleng.2006.03.002.

Tratalos, J, R A Fuller, K L Evans, R G Davies, S E Newson, J J D Greenwood, and K J Gaston. 2007. Bird densities are associated with household densities. *Global Change Biology* 13: 1685–1695.

Tratalos, J, R A Fuller, P H Warren, R G Davies, and K J Gaston. 2007. Urban form, biodiversity potential and ecosystem services. *Landscape and urban Planning* 83: 308–317.

Troy, A, and M A Wilson. 2006. Mapping ecosystem services: Practical challenges and opportunities in linking GIS and value transfer. *Ecological Economics* 60: 435–449.

Turner, R K, L Lefler, and B Freedman. 2005. Plant communities of selected urbanized areas of Halifax, Nova Scotia, Canada. *Landscape and urban Planning* 71: 191–206.

Tyrväinen, L. 2001. Economic valuation of urban forest benefits in Finland. *Journal of environmental management* 62: 75–92. doi:10.1006/jema.2001.0421.

Tyrvainen, Liisa. 2000. Property Prices and Urban Forest Amenities. *Journal of Environmental Economics and Management* 39: 205–223.

Upmanis, Hillevi, and Deliang Chen. 1999. Influence of geographical factors and meteorological variables on nocturnal urban-park temperature differences — a case study of summer 1995 in Göteborg , Sweden. *Climate Research* 13: 125–139.

Vandermeulen, Valerie, Ann Verspecht, Bert Vermeire, Guido Van Huylenbroeck, and Xavier Gellynck. 2011. The use of economic valuation to create public support for green infrastructure investments in urban areas. *Landscape and Urban Planning* 103. Elsevier B.V. 198–206. doi:10.1016/j.landurbplan.2011.07.010.

Vrscaj, B, L Poggio, and F A Marsan. 2008. A method for soil environmental quality evaluation for management and planning in urban areas. *Landscape and urban Planning* 88: 81–94.

Walker, Jason S, Nancy B Grimm, John M Briggs, Corinna Gries, and Laura Dugan. 2009. Effects of urbanization on plant species diversity in central Arizona. *Frontiers in Ecology and the Environment* 7: 465–470. doi:10.1890/080084.

Wallace, Ken J. 2007. Classification of ecosystem services: Problems and solutions. *Biological Conservation* 139: 235–246. doi:10.1016/j.biocon.2007.07.015.

Whitford, V, A R Ennos, and J F Handley. 2001. “City form and natural process” - indicators for the ecological performance of urban areas and their application to Merseyside; UK. *Landscape and Urban Planning* 57: 91–103.

Xiao, Qingfu, Gregory E McPherson, Susan L Ustin, Mark E Grismer, and James R Simpson. 2000. Winter rainfall interception by two mature open-grown trees in Davis , California. *Hydrological Processes* 14: 763–784.

Yadav, Priyanka, Kathy Duckworth, and Parwinder S. Grewal. 2012. Habitat structure influences below ground biocontrol services: A comparison between urban gardens and vacant lots. *Landscape and Urban Planning* 104. Elsevier B.V. 238–244. doi:10.1016/j.landurbplan.2011.10.018.

Yang, Jun, Joe Mcbride, Jinxing Zhou, and Zhenyuan Sun. 2005. The urban forest in Beijing and its role in air pollution reduction. *Urban Forestry & Urban Greening* 3: 65–78. doi:10.1016/j.ufug.2004.09.001.

Yang, Jun, Qian Yu, and Peng Gong. 2008. Quantifying air pollution removal by green roofs in Chicago. *Atmospheric Environment* 42: 7266–7273. doi:10.1016/j.atmosenv.2008.07.003.

Yang, Xiaojun. 2012. An assessment of landscape characteristics affecting estuarine nitrogen loading in an urban watershed. *Journal of environmental management* 94. Elsevier Ltd: 50–60. doi:10.1016/j.jenvman.2011.07.025.

Yin, Shan, Zhemin Shen, Pisheng Zhou, Xiaodong Zou, Shengquan Che, and Wenhua Wang. 2011. Quantifying air pollution attenuation within urban parks: An experimental approach in Shanghai, China. *Environmental Pollution* 159: 2155–2163.

Yu, Chen, and Wong Nyuk Hien. 2006. Thermal benefits of city parks. *Energy and Buildings* 38: 105–120. doi:10.1016/j.enbuild.2005.04.003.

Yuan, F. 2008. Land-cover change and environmental impact analysis in the Greater Mankato area of Minnesota using remote sensing and GIS modelling. *International Journal of Remote Sensing* 29: 1169–1184. doi:10.1080/01431160701294703.

Zerbe, S, U Maurer, S Schmitz, and H Sukopp. 2003. Biodiversity in Berlin and its potential for nature conservation. *Landscape and Urban Planning* 62: 139–148.

Zhang, Yan, Zhifeng Yang, and Wei Li. 2006. Analyses of urban ecosystem based on information entropy. *Ecological Modelling* 197: 1–12. doi:10.1016/j.ecolmodel.2006.02.032.

Zhao, B, U Kreuter, B Li, Z Ma, J Chen, and N Nakagoshi. 2004. An ecosystem service value assessment of land-use change on Chongming Island, China. *Land Use Policy* 21: 139–148.

Zhou, Yuan, Tiemao Shi, Yuanman Hu, Chang Gao, Miao Liu, Shilei Fu, and Shizhe Wang. 2011. Urban green space planning based on computational fluid dynamics model and landscape ecology principle: A case study of Liaoyang City, Northeast China. *Chinese Geographical Science* 21: 465–475. doi:10.1007/s11769-011-0488-7.

List of reviews not included in the study

Colding, Johan, Jakob Lundberg, and Carl Folke. 2006. Incorporating green-area user groups in urban ecosystem management. *Ambio* 35: 237–44.

De Groot, R.S., R. Alkemade, L. Braat, L. Hein, and L. Willemen. 2010. Challenges in integrating the concept of ecosystem services and values in landscape planning, management and decision making. *Ecological Complexity* 7. Elsevier B.V. 260–272. doi:10.1016/j.ecocom.2009.10.006.

De Groot, R S, M A Wilson, and R M J Boumans. 2002. A typology for the classification, description and valuation of ecosystem functions, goods and services: Special Issue: The Dynamics and Value of Ecosystem Services: Integrating Economic and Ecological Perspectives. *Ecological Economics* 2002: 393–408.

Ernstson, Henrik, Stephan Barthel, Erik Andersson, and Sara T Borgström. 2010. Scale-Crossing Brokers and Network Governance of Urban Ecosystem Services : The Case of Stockholm 15.

Hermann, Anna, Sabine Schleifer, and Thomas Wrbka. 2011. The Concept of Ecosystem Services Regarding Landscape Research : A Review. *Living Reviews in Landscape Research* 5: 1–37.

McComb, G, V Lantz, K Nash, and R Rittmaster. 2006. International valuation databases: Overview, methods and operational issues. *Ecological Economics* 60: 461–472.

Nijkamp, Peter, Gabriella Vindigni, and Paulo a.L.D. Nunes. 2008. Economic valuation of biodiversity: A comparative study. *Ecological Economics* 67: 217–231. doi:10.1016/j.ecolecon.2008.03.003.

Price, Colin. 2003. Quantifying the aesthetic benefits of urban forestry. *Urban Forestry & Urban Greening* 1: 123–133.

Swinton, S M, F Lupi, G P Robertson, and S K Hamilton. 2007. Ecosystem services and agriculture: Cultivating agricultural ecosystems for diverse benefits. *Ecological Economics* 64: 245–252.

Von Haaren, Christina, and Christian Albert. 2011. Integrating ecosystem services and environmental planning: limitations and synergies. *International Journal of Biodiversity Science, Ecosystem Services & Management*: 1–18. doi:10.1080/21513732.2011.616534.

Cameron, Ross W.F., Tijana Blanuša, Jane E. Taylor, Andrew Salisbury, Andrew J. Halstead, Béatrice Henricot, and Ken Thompson. 2012. The domestic garden – Its contribution to urban green infrastructure. *Urban Forestry & Urban Greening* 11: 129–137. doi:10.1016/j.ufug.2012.01.002.
